# Early ^18^F-FDG PET/CT Evaluation Shows Heterogeneous Metabolic Responses to Anti-EGFR Therapy in Patients with Metastatic Colorectal Cancer

**DOI:** 10.1371/journal.pone.0155178

**Published:** 2016-05-19

**Authors:** Erik J. van Helden, Otto S. Hoekstra, Ronald Boellaard, Chantal Roth, Emma R. Mulder, Henk M. W. Verheul, C. Willemien Menke-van der Houven van Oordt

**Affiliations:** 1 VUmc Cancer Center Amsterdam, VU University medical center, Department of Medical Oncology, Amsterdam, the Netherlands; 2 Department of Radiology and Nuclear Medicine, VU University Medical Center, Amsterdam, the Netherlands; Catalan Institute of Oncology, SPAIN

## Abstract

**Objective:**

The aim of this pilot study was to explore intrapatient mixed metabolic response and early ^18^F-FDG PET response evaluation using predefined quantification strategies in patients with advanced KRAS wild-type colorectal adenocarcinoma (mCRC) treated with cetuximab.

**Methods:**

A ^18^F-FDG PET was performed at baseline and after 2 cycles of cetuximab. Metabolic response was categorized using thresholds suggested in PERCIST. Quantitative analysis was done for the sum of all target lesions, ≤ 5 lesions and the metabolically most active lesion per PET. Quantitative data were correlated with clinical benefit, according to RECIST v1.1, after two months of treatment.

**Results:**

In nine evaluable patients the total number of target lesions was 34 (1–8 per patient). Mixed metabolic response was observed in three out of seven patients with multiple target lesions, using TLG. Dichotomised metabolic data of the sum of all or ≤ 5 lesions had a concordance with clinical benefit of 89% using SUL_max_ or SUL_peak,_ and 100% using TLG. Evaluating the metabolically most active lesion, concordance was 89% for all three units. Additionally, the decrease in TLG was significantly correlated with PFS for all three quantification strategies.

**Conclusion:**

Mixed metabolic response was observed in nearly half of the patients with advanced KRAS wild-type mCRC treated with cetuximab. If ≤ 5 target lesions were evaluated using TLG clinical benefit was predicted correctly for all patients. Moreover, decrease in TLG is significantly correlated with the duration of PFS. Validation of these promising preliminary results in a larger cohort is currently on-going.

**Trial Registration:**

ClinicalTrials.gov NCT01691391

## Introduction

Early changes in glucose metabolism defined with ^18^F-fluorodeoxyglucose positron emission tomography / computed tomography (^18^F-FDG PET) is a potential tool to differentiate between responders and non-responders early after start of anti-cancer treatment [1–5]. The advantage of ^18^F-FDG PET compared to anatomic evaluation of tumour lesions is that changes in metabolic activity can be assessed shortly after start of therapy [4], whereas anatomic evaluation can only be performed after 2–3 months of treatment.

Complete visual resolution of radiotracer uptake is fairly straightforward and demonstrated to be a good prognostic marker [6]. Yet, with early response evaluation, especially for treatment with targeted agents like cetuximab, smaller changes in ^18^F-FDG uptake are expected, even in responding patients. Consequently, comparability of acquisition, quantification and response criteria are crucial for the efficacy of ^18^F-FDG PET as early response marker. The initial ^18^F-FDG PET response criteria [7] do not specify strategies for patients with multiple lesions. In clinical practice and in trials, response to therapy is typically classified at a patient level. In 2009 Wahl et al. proposed the PERCIST guideline [8], in which response is classified using the lesion with the highest radiotracer uptake per time-point, hypothesizing that this lesion is prognostically most relevant. Other strategies are to evaluate changes in ^18^F-FDG uptake for the sum of all lesions, or for the sum of ≤ 5 lesions (as in Response Evaluation Criteria In Solid Tumours (RECIST) version 1.1 for anatomic evaluation). The quantification strategy for multiple target lesions may be crucial for patients with a heterogeneous response between tumour lesions. Moreover, mixed metabolic response and the effect on response prediction may be particularly relevant for targeted agents, as response of individual lesions may be correlated with the variation of expression of the target or the presence of a resistance-inducing mutation between lesions. Additionally, the optimal type of quantification unit for response prediction, such as standardized uptake value for lean body mass (SUL)_max_, SUL_peak_ or total lesion glycolysis (TLG), remains unclear.

Early response evaluation for mCRC patients treated with anti-EGFR therapy is clinically relevant as only half of the patients will have clinical benefit [9–11]. Since there are no other known biomarkers, all patients with RAS wild-type mCRC will receive this treatment until first CT evaluation after 2–3 months (4–7 cycles). By identifying non-responders after just one or two cycles, exposition to ineffective drugs can be avoided and other treatment options can be considered. To our knowledge no metabolic data other than a case report [12] have been published regarding early response evaluation in patients treated with anti-EGFR antibody monotherapy.

The aim of this pilot study was to investigate intrapatient mixed metabolic response based on early response evaluation with ^18^F-FDG PET after 2 cycles of cetuximab monotherapy in patients with KRAS wild type mCRC. Additionally, the impact of mixed metabolic response on three quantification strategies (the sum of all target lesions, the sum of ≤ 5 lesions and the metabolically most active lesion) with three quantitative PET metrics (SUL_max_, SUL_peak_ and TLG) were evaluated. Metabolic parameters were correlated with standard CT evaluation according to RECIST v1.1.

## Patients and Methods

### Patients

Patients with unresectable KRAS wild-type mCRC who had been treated according to standard care (fluoropyrimidines, oxaliplatin and irinotecan) and were candidates for anti-EGFR antibody monotherapy were eligible for this PET imaging study with ^89^Zr-cetuximab [13] and ^18^F-FDG PET. All in- and exclusion criteria are stated in S1 Table. At the time of patient accrual, RAS mutations in KRAS exon 3 and 4 and NRAS exon 2, 3 and 4 were not yet identified as biomarkers for primary resistance, therefore only wild type KRAS exon 2 was required for inclusion. Additional RAS and BRAF (exon 15) mutations were analysed retrospectively. Patients were treated biweekly with cetuximab 500mg/m^2^. This single centre, two step non-randomized intervention study was reviewed and approved (d.d. 27-06-2011) by the Ethics Committee at VU University Medical Center (VUmc) before the study opened for inclusion. All patients signed informed consent before any study activities were conducted. The authors confirm that all ongoing and related trials for this drug/intervention are registered (clinicaltrials.gov NCT01691391; d.d. 15-05-2012; S1 and S2 Files).

### ^18^F-FDG PET/CT

^18^F-FDG PET/CT’s were performed at baseline (within two weeks prior to the first cetuximab infusion) and after four weeks of treatment. All ^18^F-FDG PET were conducted according to European Association of Nuclear Medicine guidelines [14]. Briefly, patients fasted 6 hours prior to the radiotracer injection. Patients were injected with 3 MBq/kg (± 10%) ^18^F-FDG. After 60 min (± 5 min) a PET scan was performed from skull base to mid-thigh. Residual activity in the syringe was measured and subtracted from the injected dose before calculations were done.

Data-analysis of all ^18^F-FDG PET was done after all data was collected and follow-up ended (January 2015). Target lesions were defined as tumour lesions with a minimal diameter of ≥ 2 cm on CT scan, to minimize potential partial volume effect, and with an uptake above background activity. The background activity was calculated in healthy liver tissue in a 3x3 cm Volume Of Interest (VOI) (using the following formula: (1.5x average SUL liver) + (2x SD average SUL liver)) or in the descending aorta in a 1x1 cm VOI (using the following formula: (2x average SUL descending thoracic aorta) + (2x SD average SUL aorta) in case of hepatic metastases according to PERCIST [8]. Tumour VOI’s were created using a semi-automatic delineation tool which used a 50% standardized uptake value (SUV)_max_ threshold with background correction [15]. If semi-automatic delineation was not possible (mostly due to tissue with high uptake positioned closely to the target lesion), the VOI’s were created manually and shrunk down using the same 50% SUV_max_ threshold with background correction.

Three quantification units were evaluated: SUL_peak_ (derived from the activity measured in a 1 cm^3^ sphere within the tumour VOI, placed automatically to ensure that it captured the highest mean radioactivity), SUL_max_ (defined as the voxel with the most radioactivity within the tumour VOI), and TLG (defined as SUL_mean_ times metabolically active tumour volume, where this volume was defined with an isocontour VOI of 50% of SUV_peak_ with background correction) [16]. Quantitative analysis of the PET images was performed using three different strategies: 1. all target lesions with a minimal diameter of 2 cm, evaluated separately (paired for the 2 time points) and summed, 2. the sum of ≤ 5 (≤ 2 per organ) metabolically most active target lesions per PET scan (as is suggested in PERCIST as exploratory approach and as is used for anatomical changes according to RECIST version 1.1 [17]), 3. the single most metabolically active lesion per PET scan (baseline and on-treatment target lesion can differ, c.f. PERCIST).

Metabolic data were classified using predefined criteria according to PERCIST as complete metabolic response (CMR), partial metabolic response (PMR, SUL reduction of ≥ 30% and ≥ 0.8 unit; TLG reduction of ≥ 45%), stable metabolic disease (SMD, changes <30% range for SUL and < 45% range for TLG) and progressive metabolic disease (PMD, increase of ≥ 30% SUL and ≥ 0.8 unit; TLG increase of ≥ 45%) [8]. A patient was classified with mixed metabolic response if lesions (from paired metabolic data) within one patient were categorized in different response categories (e.g. CMR, PMR, SMD and PMD as mentioned above).

Since the aim of early response evaluation is the differentiation between patients with and without clinical benefit, metabolic data was dichotomized as metabolic response (reduction of SUL with ≥ 30% and ≥ 0.8 unit; TLG reduction of ≥ 45%) and metabolic non-response (a reduction smaller than the previously mentioned limits or an increase in ^18^F-FDG uptake). Additionally, progression free survival (PFS) was correlated with the percentage change in ^18^F-FDG uptake using Spearman's rank correlation coefficient, this test was performed using SPSS version 22 (IBM Corp., Armonk, NY). A p value < 0.05 was considered as significantly relevant.

### CT analysis

During treatment with cetuximab, diagnostic computed tomography (CT) scans were performed every 8 weeks until progressive disease or discontinuation of treatment. Response was evaluated and categorised using RECIST version 1.1 as progressive disease (PD), stable disease (SD), partial response (PR) or complete response (CR) [17]. Clinical benefit is defined as SD, PR or CR as best response to treatment. PFS is defined as the period starting at the first treatment until PD. At the time of publication all patients had progressive disease according to RECIST. A physician, blinded for ^18^F-FDG-PET data, defined the RECIST target lesions.

Apart from the RECIST measurements, all tumour lesions ≥ 2 cm were measured separately (paired) to study heterogeneity in response and correlate with metabolic changes. These anatomic changes for single lesions were categorized using the same thresholds as in RECIST.

## Results

**Fig 1:**
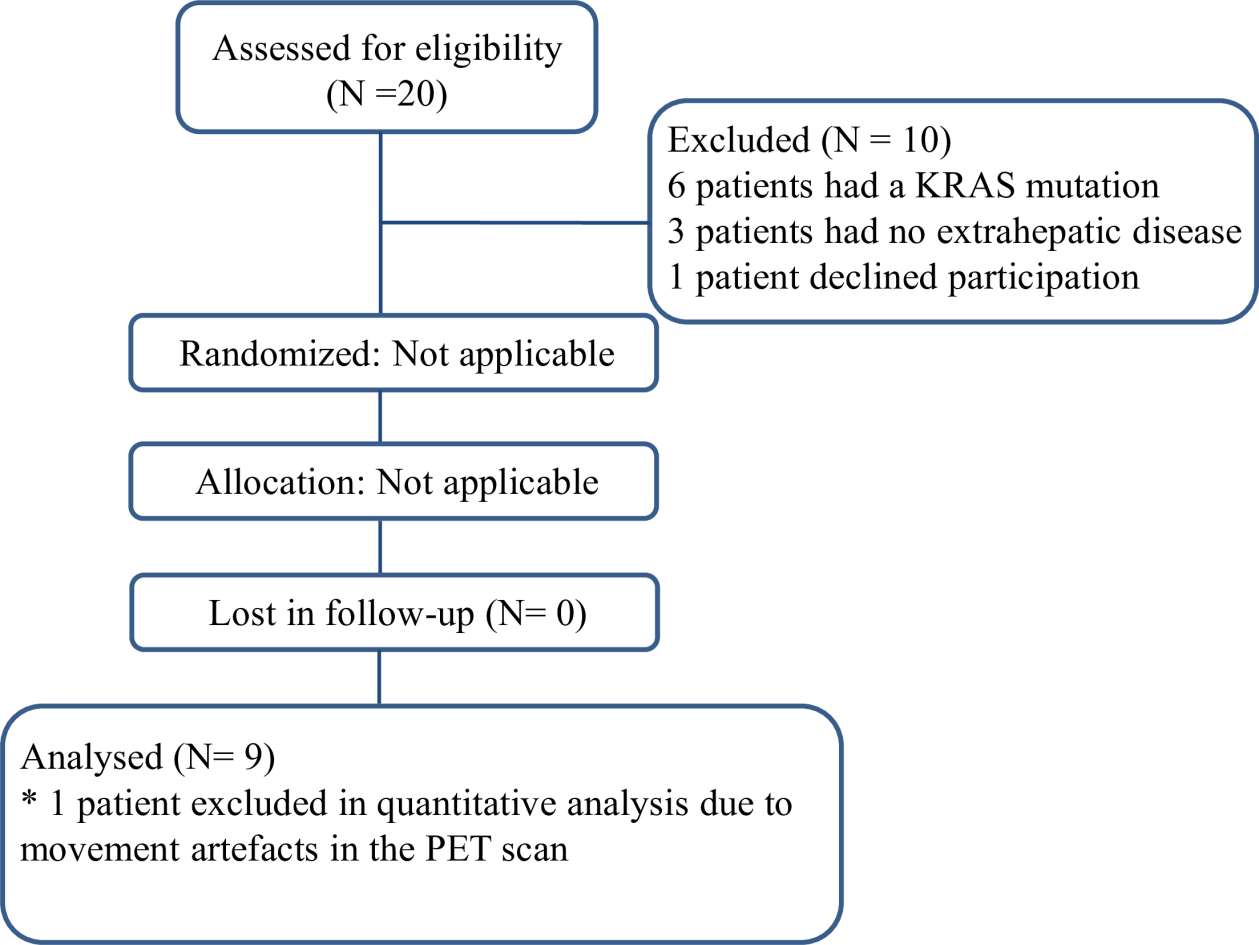
CONSORT flow-chart.

Out of 20 patients screened at VUmc, 10 were not eligible (60% KRAS mutation, 30% no extra-hepatic disease, 10% declined participation) and the remaining patients were included (Fig 1). The study was open for inclusion until March 2014, follow-up ended in January 2015, all patients had progressive disease at that time. There were no patients lost in follow-up. At the time of inclusion patients had a median age of 61 years (with a range 50–73 years), 60% were male and the majority of patients had an adenocarcinoma of the rectum or sigmoid (S2 Table). All patients were KRAS (exon 2 / 3) wild-type, in half of the patients additional RAS mutations were tested and proven wild-type. For the other five patients, the tumour tissue quality was not sufficient for retrospective mutation analysis. All patients were tested for mutations in BRAF exon 15, only patient number 5 had this prognostically poor mutation. Five patients had PD at the first CT evaluation (after 8 weeks of treatment), two had PR and three had SD according to RECIST. Median PFS was 8 weeks, with a range of 6–33 weeks (S2 Table).

Due to movement artefacts one patient (#1) was excluded for quantitative analyses. The remaining patients had 34 target lesions with a diameter of ≥ 2 cm and ^18^F-FDG uptake above background, with a range of 1 to 8 target lesions per patient. Most target lesions were located in the liver, adrenal glands, lungs or lymph nodes.

The background radioactivity was measured in the liver for three patients and for six patients with hepatic metastases this was done in the descending aorta. Three patients had ≥ 20% difference in background activity between baseline and on-treatment PET. One patient had an increased liver uptake of 21% (+0.50 unit), most likely due to a protocol deviation consisting of a longer interval between ^18^F-FDG administration and PET scanning at baseline. Nevertheless, this patient had an increased accumulation of ^18^F-FDG in target lesions at the on-treatment PET (i.e. PMD), concordant with the first evaluation CT scan which showed PD. Two patients had a decrease of 25% (-0.60 and -0.63 unit) in SUL_mean_ in the background activity of the aorta. However, in healthy liver the difference did not surpass the 20% threshold.

### Mixed metabolic response

**Fig 2:**
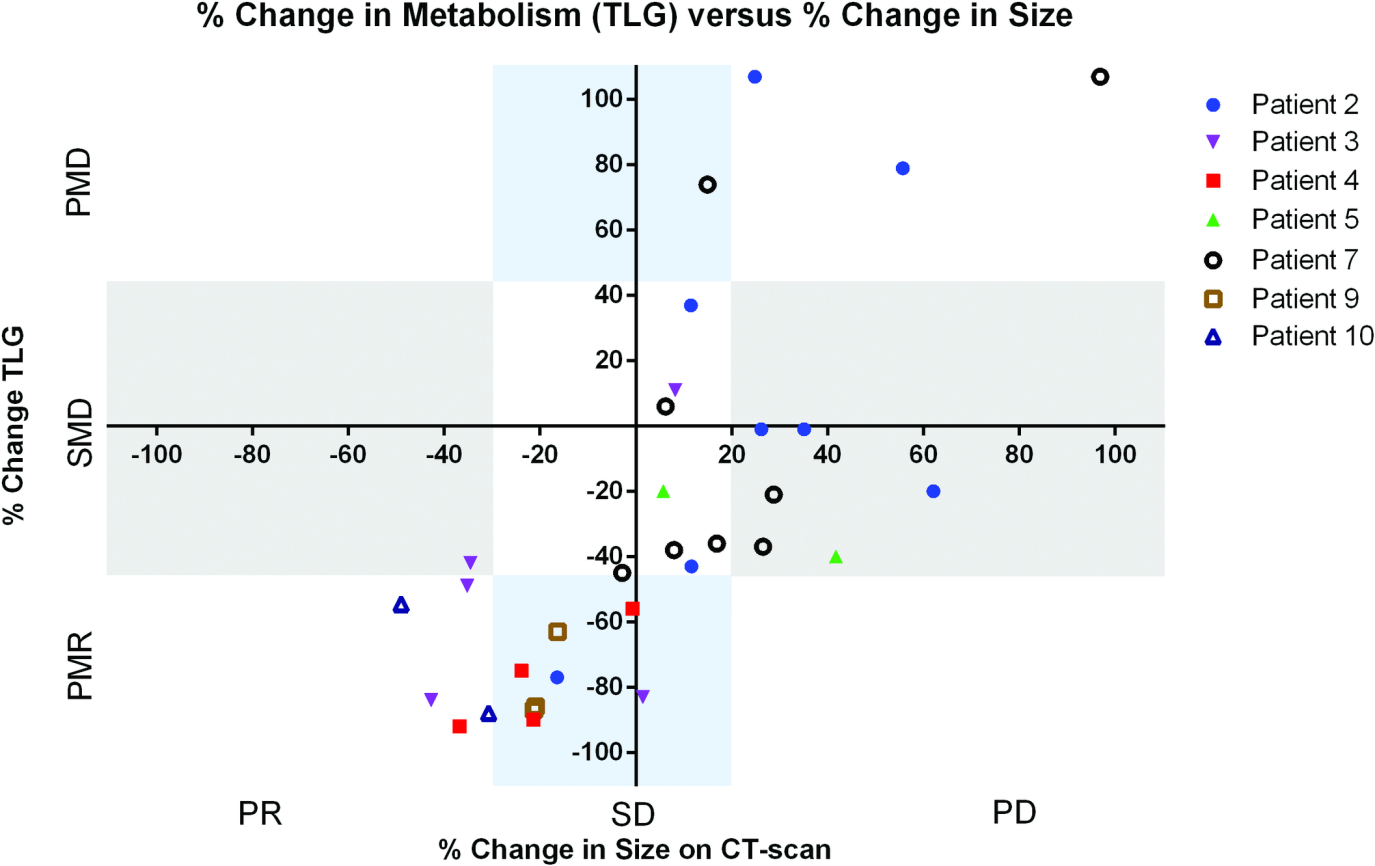
Percentage change in tumour size on CT-scan after 2 months of treatment (x-axis) is compared with percentage change in ^18^F-FDG uptake in TLG after 4 weeks of treatment (y-axis) for each separate tumour lesion (bone lesions, being non-measurable lesions, are excluded in this graph). A clear trend is noticeable between change in ^18^F-FDG uptake and change in size. Intrapatient mixed metabolic response is most pronounced in patient 2; different lesions within this patient are categorized as PMR, SMD and PMD. Patient 3 has lesions in both PMR and SMD categories and patient 7 have lesions in both SMD and PMD categories. In all patients minor heterogeneity in anatomic changes is observed.

To evaluate intrapatient heterogeneous metabolic response between tumour lesions, changes in ^18^F-FDG uptake after 4 weeks of treatment (2 cycles of cetuximab) and anatomic changes on first CT evaluation (after 8 weeks, i.e. 4 cycles of treatment) were compared for all tumour target lesions separately. Of the nine evaluable patients, seven had multiple target lesions. Intrapatient mixed metabolic response was observed in three out of seven patients when tumour uptake was expressed in TLG. Heterogeneity was most pronounced in patient 2, with metabolically responding, stable and progressive target lesions. Additionally, some heterogeneity in TLG data was observed in patient 3 and 7 (Fig 2). Using SUL_peak_, intrapatient mixed metabolic response was observed in patient 2 and 3, for SUL_max_ mixed response was observed in patient 2 and 7 (S1 and S2 Figs).

Anatomic changes on CT scan of all target lesions (bone lesions, being non-measurable lesions, are excluded) demonstrated minor heterogeneity; different lesions of one patient could be categorized as PR and SD or as PD and SD, but never both progressive and responding lesions within one patient. There was a weak trend between decrease of ^18^F-FDG uptake and that of tumour lesion size (Fig 2). All ten tumour lesions demonstrating anatomic growth at week 8 had stable or progressive metabolic disease at week 4. Five out of six tumour lesions with partial response based on anatomic size had partial metabolic response. For anatomically stable lesions all three metabolic response criteria were observed.

### Early response evaluation: the sum of all target lesions

**Fig 3:**
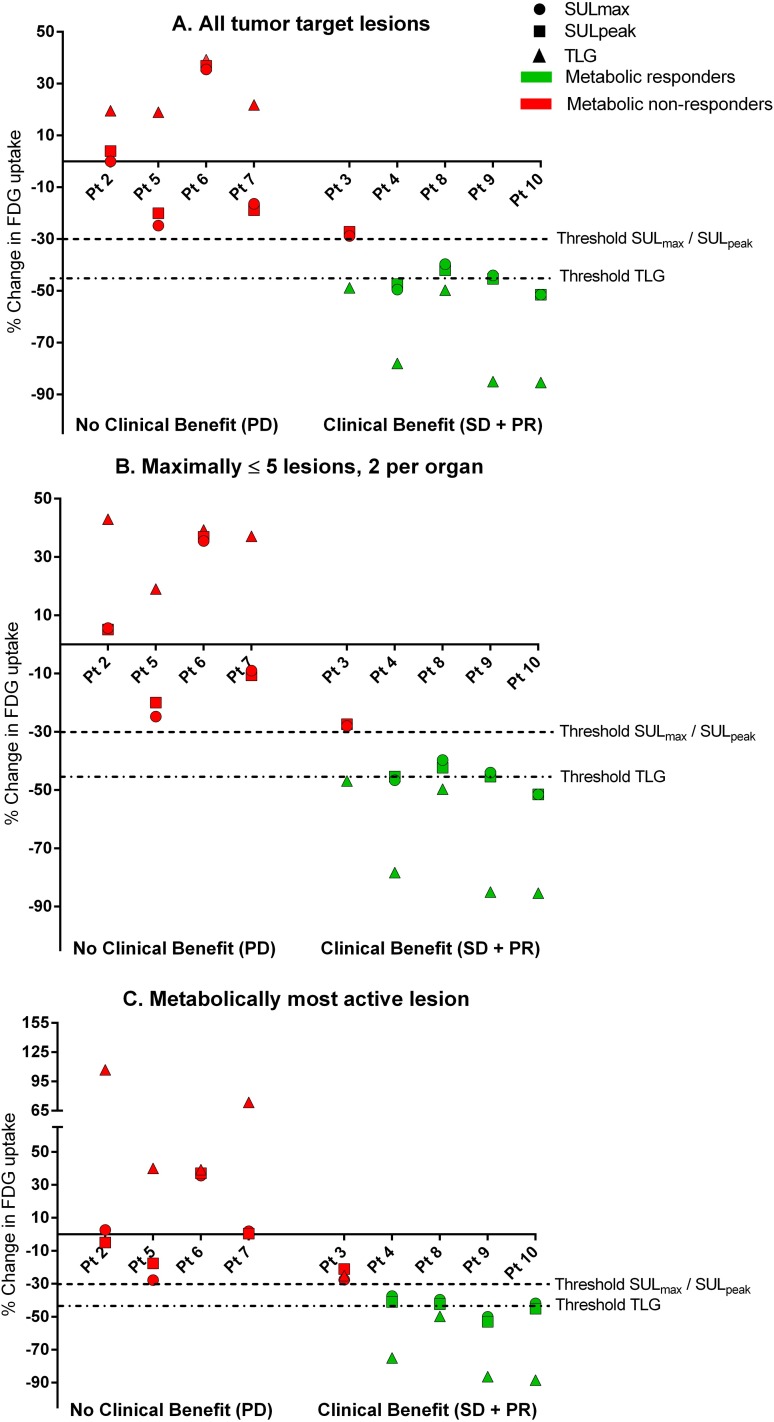
Panel A illustrates percentage change in ^18^F-FDG uptake for the sum of all tumour lesions expressed in SUL_max_, SUL_peak_ and TLG. Patients without clinical benefit (left side) are all correctly categorised as metabolic non-responders. If uptake is expressed in TLG response prediction was accurate for all patients. For SUL_max_ and SUL_peak_ patient 3 who had clinical benefit, was miscategorised as metabolic non-responder. Panel 3B illustrates percentage change in ^18^F-FDG uptake for the sum of ≤ 5 lesions (≤ 2 per organ) per PET scan. Metabolic response categories are identical to response prediction based on the sum of all target lesions. Panel **3**C illustrates percentage change in ^18^F-FDG uptake of the metabolically most active lesion per PET scan. Patient 3 is miscategorised as metabolic non-responders for all three quantification units.

In Fig 3A the percentage change of the sum of all target lesions is depicted. Metabolic data is dichotomized into metabolic responders (decreased of ≥ 30% SUL or ≥ 45% TLG), or metabolic non-responders (an increase or decrease under the response limit), since this resulted in the best discrimination between patients with and without clinical benefit according to RECIST. Expressing changes in ^18^F-FDG uptake using SUL_max_ and SUL_peak_ resulted in an 89% concordance with clinical benefit (S3 Fig). However, with TLG early response prediction is accurate for all patients.

**Fig 4:**
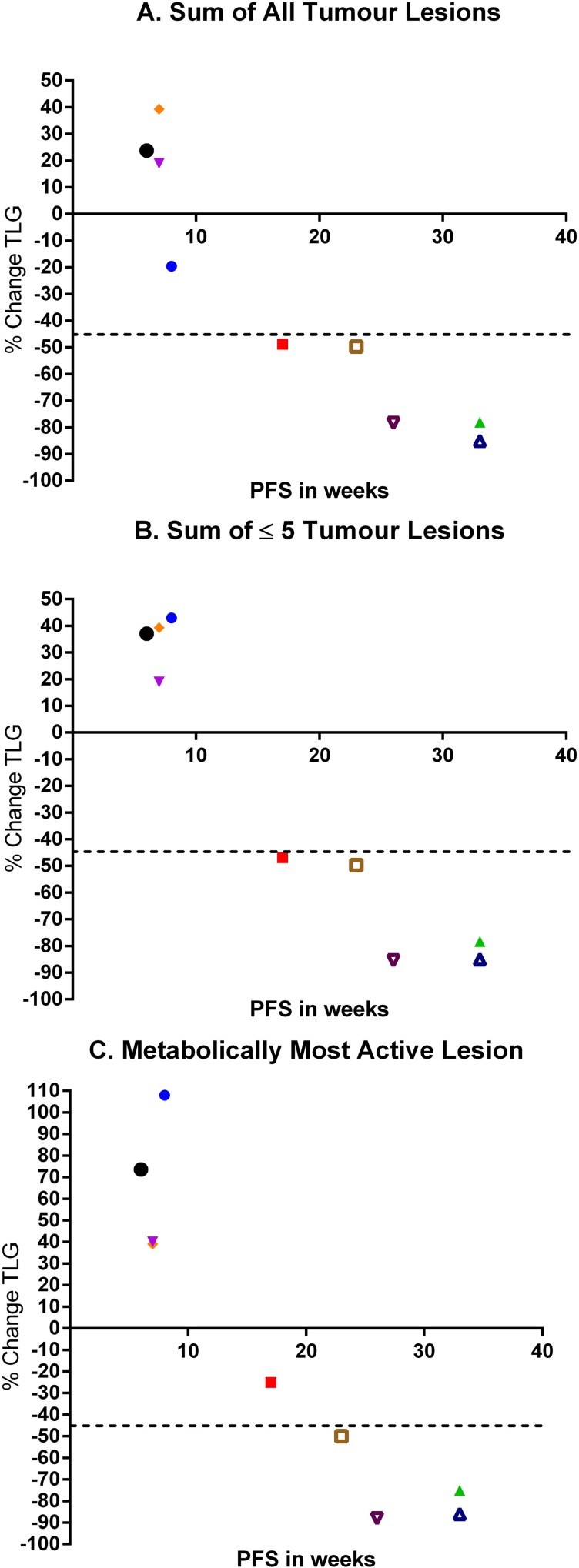
Panel A illustrates the percentage change of the sum of all target lesions expressed in TLG versus PFS in weeks. All patients with clinical benefit have a > 45% decrease in TLG. Notably, there is a significant correlation between reduction in TLG and a longer PFS (R_s_ -0.94, p-value < 0.001). Panel B illustrates the percentage change of the sum of ≤ 5 target lesions expressed in TLG versus PFS in weeks. Again there is a significant correlation between ^18^F-FDG decrease and the duration of PFS (R_s_ -0.82, p-value = 0.009). Panel C illustrates the percentage change of the metabolically most active lesion expressed in TLG versus PFS in weeks (R_s_ -0.84, p-value = 0.006). Patient number 3 was misclassified as non-responder (red square above dotted line). Yet, this patient fits the line, with a moderate decrease in TLG and a relative short PFS.

Besides the correlation between metabolic response and clinical benefit, metabolic data of the sum of all target lesions is compared to the duration of PFS. As illustrated in Fig 4A, there is a significant correlation (Spearman's rank correlation coefficient; R_s_ -0.94, p-value < 0.001) between the percentage decrease of the sum of all target lesions expressed in TLG after 4 weeks and the duration of PFS. Also for ≤ 5 lesions and the metabolically most active lesion there is a significant correlation (Fig 4B and 4C; R_s_ -0.82, p-value = 0.009 and R_s_ -0.84, p-value = 0.006 respectively).

### Early response evaluation: the sum of ≤ 5 target lesions

In Fig 3B, the percentage change of the sum of ≤ 5 lesions (≤ 2 lesions per organ) is depicted. Patients 2, 3, 4 and 7 had > 5 target lesions. Early response prediction based on ≤ 5 target lesions rendered identical response categories for all patients compared to response prediction based on the sum of all target lesions, for all three quantification units. A 100% concordance with clinical benefit is achieved when ^18^F-FDG uptake is expressed in TLG. As with response prediction based on all lesions, changes in ^18^F-FDG based on ≤ 5 target lesions demonstrated a clear trend between ^18^F-FDG decrease and duration of PFS (Fig 4B).

### Early response evaluation: the metabolically most active lesion (cf. PERCIST)

**Fig 5:**
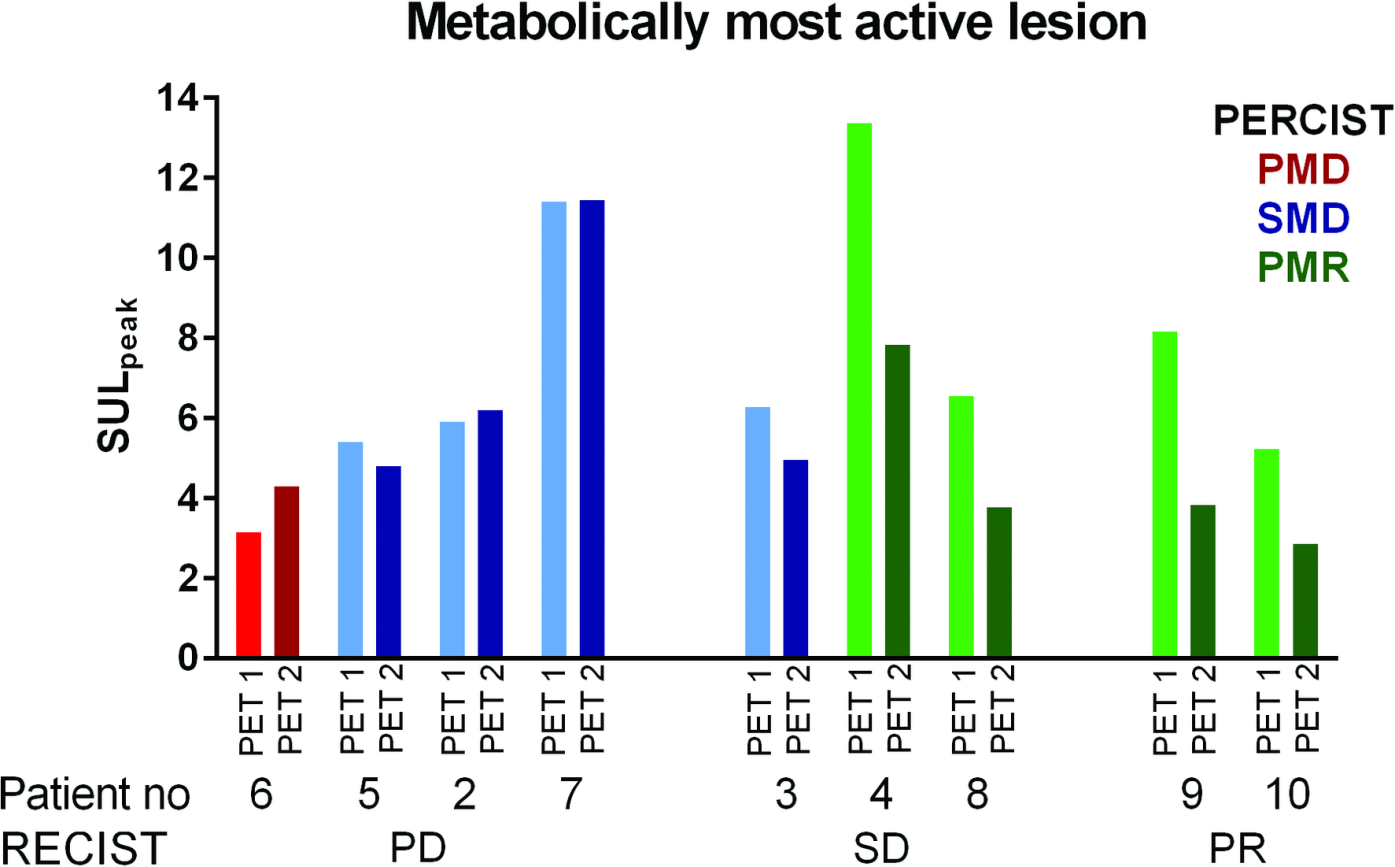
A comparison between RECIST and PERCIST categories. Patients are grouped on the x-axis based on RECIST, with different colours to indicate the PERCIST categories. On the y-axis change in SUL_peak_ of the baseline and on-treatment ^18^F-FDG PET is demonstrated. The majority of patients which had PD based on CT evaluation had SMD. Of the three patients who had prolonged stable disease, two patients had PMR and one patient had SMD. Both patients with PR based on CT evaluation had PMR on the early response evaluation with ^18^F-FDG PET.

Three patients had a different target lesion at baseline PET compared to the on-treatment PET using the metabolically most active lesion per time point. Concordance between PERCIST (using SUL_peak_) and RECIST response categories was 44% (Fig 5). However, dichotomized metabolic data correctly predicted clinical benefit in 89% (Fig 3C), misclassifying patient 3 as metabolic non-responder, with a decrease of 21% in SUL_peak_. The metabolically most active lesion per scan expressed in SUL_max_ and TLG demonstrated an identical distribution as with SUL_peak_ (Fig 3C), again misclassifying patient 3 with a decrease of 28% and 25% using SUL_max_ and TLG, respectively (all data was summarised in S3 Table).

## Discussion

In this study mixed metabolic response was observed in three out of seven patients (43%) with multiple metastatic target lesions, treated with monotherapy cetuximab. Dichotomised metabolic data of early ^18^F-FDG PET/CT evaluation for the metabolically most active lesion yielded a concordance with clinical benefit of 89% for all three units. The sum of all or ≤ 5 lesions had a concordance with clinical benefit of 89% using SUL_max_ or SUL_peak,_ and 100% using TLG. Additionally, the decrease in TLG was significantly correlated with PFS for all three quantification strategies.

Although it is known that mixed metabolic response to (targeted) therapy occurs [2;18], the interpretation of a heterogeneous tumour biology remains unclear. Similar to our study, Hendlisz et al. reported a high incidence of mixed metabolic response (68%) in patients with mCRC treated with chemotherapy. Multiple lesions were dichotomized into (dominantly) metabolic response versus (dominantly) metabolic non-response (non-response was defined < 15% decrease in SUV_max_). All metabolic non-responders had no response according to RECIST [2], as is the case in this study. Additionally, Hendlisz et al. evaluated mixed metabolic response and the influence on survival data in 79 mCRC patients treated with sorafenib and chemotherapy. Their conclusion was that patients that only have responding lesions have a significantly longer PFS compared to patients with a heterogeneous response (p-value < 0.001) [19], this is in concordance with our data. In contrast to the study of Hendlisz et al., our patients are treated with cetuximab monotherapy. Heterogeneity in response may be more essential for monotherapy with a targeted agent, since treatment benefit may be correlated with the variation of expression of the target between lesions or the presence of a resistance-inducing mutation. To our knowledge our study is the first to report on FDG evaluation for monotherapy with a targeted agent.

The optimal quantitative strategy for an accurate ^18^F-FDG PET early response prediction is unknown. In literature, numerous small studies regarding early ^18^F-FDG PET response evaluation for cancer treatments have been published. Unfortunately, comparability is poor due to differences in quantification methods [20–22], cancer type [1;23–25], type of therapy [19;26;27], treatment line and primary outcome [2;6;20–22;24]. The PERCIST guideline is the first to describe a systematic comparable method to evaluate patients with multiple tumour lesions, it is based on the hypothesis that the lesion with the highest radiotracer uptake per time-point is prognostically most relevant. Yet, in case of mixed metabolic response, only evaluating the metabolically most active lesion might not accurately represent the entire tumour biology. Moreover, recent publications demonstrated that early metabolic response according to PERCIST guidelines might not optimally predict response according to RECIST after 2 to 3 months of therapy [6;28;29].

An alternative quantitative strategy is the evaluation of multiple target lesions. The sum of multiple tumour lesions could even out possible tumour heterogeneity. Although this study describes a small cohort, a perfect concordance was observed between dichotomized early ^18^F-FDG response prediction based on multiple tumour lesions with uptake expressed in TLG and clinical benefit. Response evaluation based on the sum of all target lesions yielded the same results as ≤ 5 target lesions, suggesting that evaluating > 5 lesions is not required for correct response prediction.

For the three patients with mixed metabolic response in our study, early response evaluation based on the metabolically most active lesion led to a correct prediction in two patients, only patient 3 was not correctly categorized as responder. Interestingly, of all patients with clinical benefit, patient 3 had the shortest PFS of just 4 months stable disease. One might question whether this patient in fact benefited from cetuximab monotherapy with only having stable disease on one CT scan. In addition, changes in early ^18^F-FDG response evaluation might predict the length of PFS more accurately (Fig 4) than response according to RECIST for patients treated with targeted agents.

This study demonstrates that early response evaluation with ^18^F-FDG PET is a promising biomarker, especially if multiple lesions are evaluated using TLG, it was based on a small cohort. As a result, differences in response prediction between the three different strategies were based on observations in a single patient. Thus, validation in a large cohort is needed. In the IMPACT-CRC study, for which patient accrual is currently ongoing, the clinical utility of early response evaluation with ^18^F-FDG PET and ^89^Zirconium labelled cetuximab PET, for patients with mCRC treated with cetuximab, will be evaluated (NCT02117466).

Finally, with respect to the commonly used semi-quantitative PET measures (SUL_max_, SUL_peak_ and TLG): SUL_max_ is less susceptible for inter-observer variability in VOI definition, but more sensitive to background noise, as it is based on only one voxel [30]. Additionally, this unit does not reflect metabolic heterogeneity within a tumour lesion [31]. SUL_peak_ is less susceptible for noise since it comprises of 1 cm^3^ of the most active part of the tumour VOI [32]. Unlike SUL, TLG is not normalized per unit mass of tumour tissue but includes change in metabolic tumour volume [16;28]. Consequently, TLG might reflect overall tumour lesion biology more accurately compared to SUL_max_ or SUL_peak_. A potential limitation of TLG is the operator dependency in defining the metabolically active tumour volume. However, repeatability of TLG was shown to be acceptable [33]. In this small cohort, uptake of multiple target lesions expressed in TLG indeed demonstrated a better concordance with clinical benefit compared to SUL_peak_ and SUL_max_.

## Conclusion

In this pilot study interlesional mixed metabolic occurred in 43% of all patients with multiple evaluable tumour (KRAS wild-type) colorectal cancer lesions, treated with cetuximab monotherapy. Early ^18^F-FDG PET response prediction based on ≤ 5 target lesions (2 ≤ per organ) expressed in TLG resulted in a correctly predicted clinical benefit in all patients. Additionally, there was a significant correlation between the decrease in TLG and PFS. Validation of these promising preliminary results in a larger patient cohort is currently on-going.

## Supporting Information

S1 Fig Percentage change in tumour size versus change in ^18^F-FDG uptake (SUL_peak_) per tumour lesion. (TIF)

S2 Fig Percentage change in tumour size versus change in ^18^F-FDG uptake (SUL_max_) per tumour lesion. (TIF)

S3 Fig The sum of all target lesions (SUL_peak_). (TIF)

S1 File Trial Study Protocol. (PDF)

S2 File TREND Statement Checklist. (PDF)

S1 Table In- and Exclusion Criteria. (DOCX)

S2 Table Patient Characteristics. (DOCX)

S3 Table Quantitative data. (DOCX)
