# Supplementary figures and images for: Early 18F-FDG PET/CT Evaluation Shows Heterogeneous Metabolic Responses to Anti-EGFR Therapy in Patients with Metastatic Colorectal Cancer
Source: PLoS One. 2016 May 19;11(5):e0155178. doi: 10.1371/journal.pone.0155178 (PMC4873260; doi:10.1371/journal.pone.0155178)

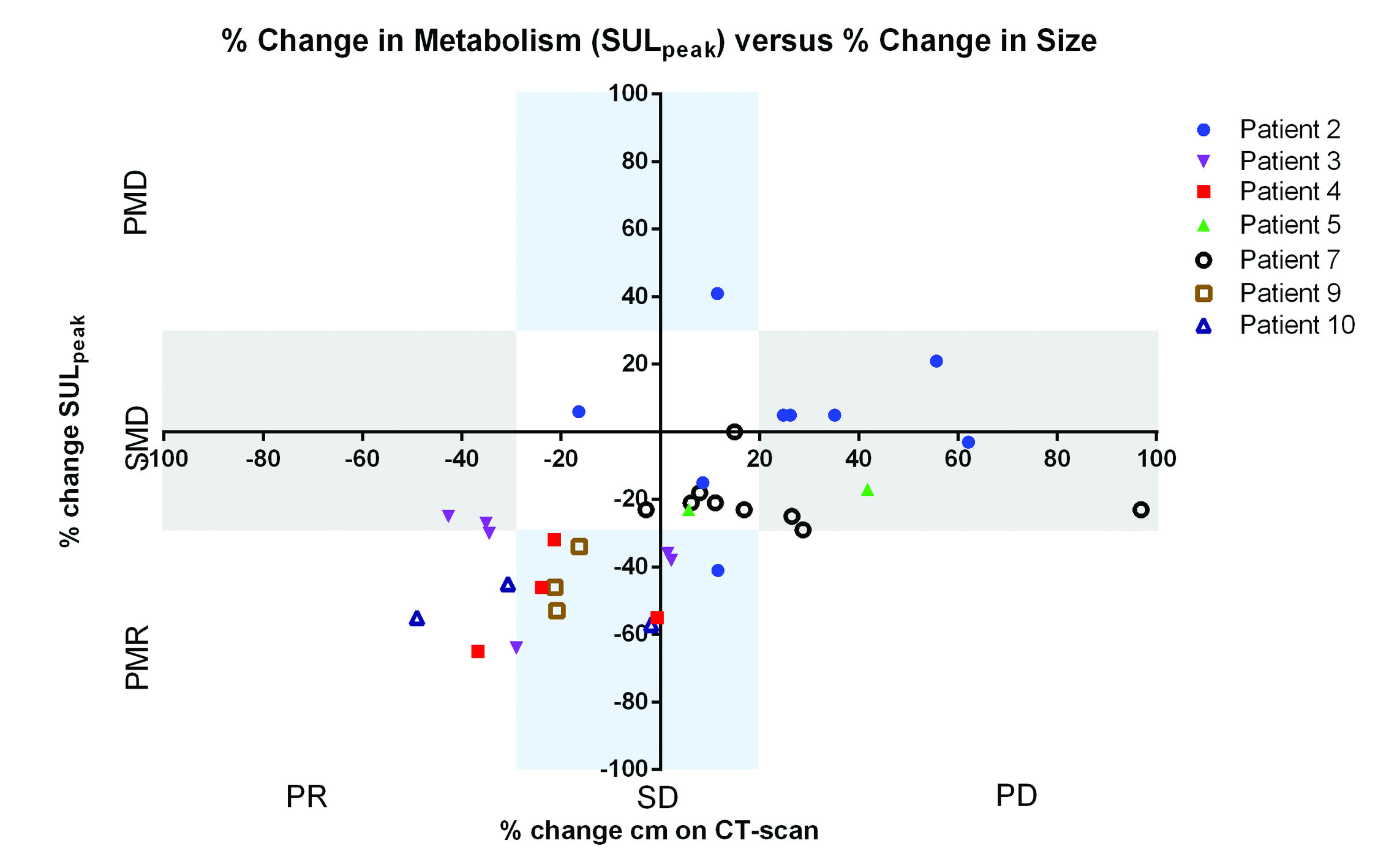

Supplement: S1 Fig — (TIF) [file pone.0155178.s001.tif]

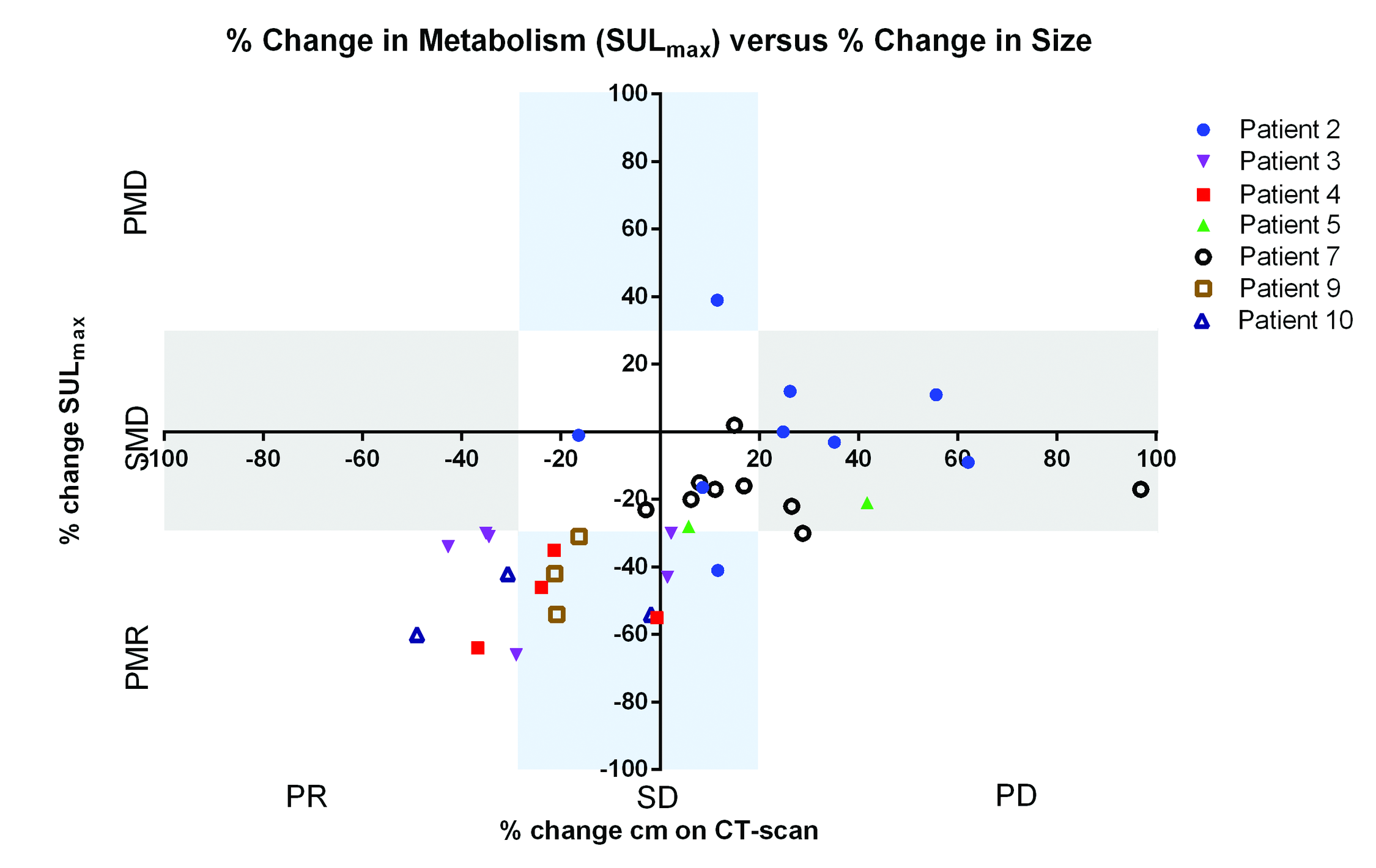

Supplement: S2 Fig — (TIF) [file pone.0155178.s002.tif]

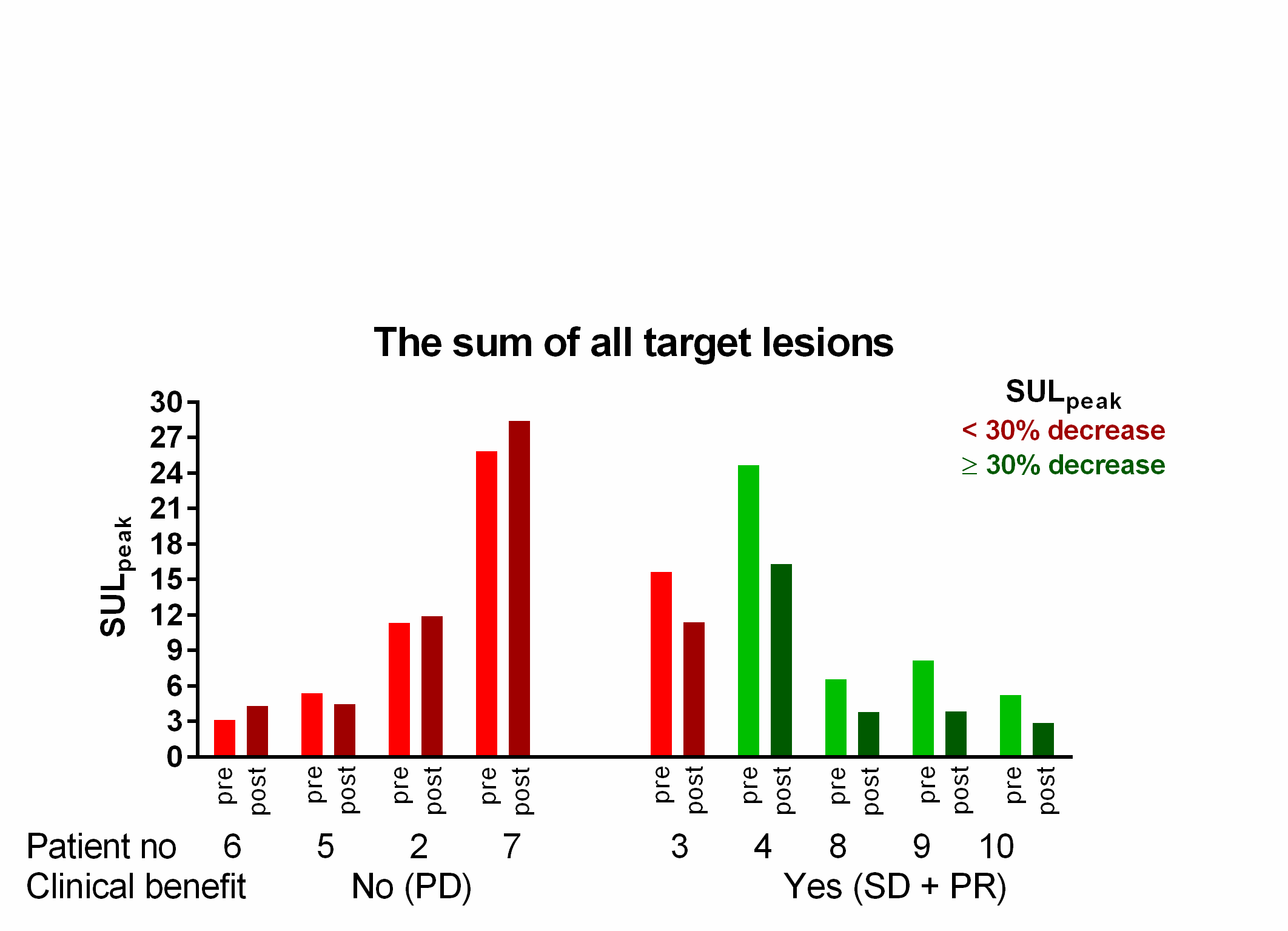

Supplement: S3 Fig — (TIF) [file pone.0155178.s003.tif]
