# Supplementary material for: Early 18F-FDG PET/CT Evaluation Shows Heterogeneous Metabolic Responses to Anti-EGFR Therapy in Patients with Metastatic Colorectal Cancer
Source: PLoS One. 2016 May 19;11(5):e0155178. doi: 10.1371/journal.pone.0155178 (PMC4873260; doi:10.1371/journal.pone.0155178)
Supplement: S1 File — (PDF) [file pone.0155178.s004.pdf]

12th joint ECCO-AACR-EORTC-ESMO Workshop 'Methods in Clinical Cancer Research'

Waldhaus Flims, Switzerland, 19 – 25 June 2010

Treatment optimization of cetuximab in patients with metastatic colorectal cancer based on tumor uptake of ^{89}Zr-labeled cetuximab assessed by PET

Protocol identification number / code / Registration n° (if applicable)

Study coordinator : Dr. CW Menke-van der Houven van Oordt, MD PhD
Department of Medical Oncology
VU University Medical Center
De Boelelaan 1117
1081 HV Amsterdam
Email: c.menke@vumc.nl
T: 020-444 4321 / F: 020-444 4079

(17-02-2011)	**Protocol approval**
(09-06-2011)	**Amendment 1**

Version: (1.0) / 05-01-2011
(2.0) / 09-06-2011

Contact addresses

Writing committee:

Prof. Dr. HMW Verheul, MD PhD
Head Department of Medical Oncology
VU University Medical Center
De Boelelaan 1117
1081 HV Amsterdam

Dr. CW Menke-van der Houven van Oordt, MD PhD
Department of Medical Oncology
VU University Medical Center

Prof Dr. GAMS van Dongen, PhD
Department of Otolaryngology/Head and Neck Surgery
VU University Medical Centre

Prof Dr. OS Hoekstra, MD PhD
Department of Nuclear Medicine & PET research
VU University Medical Centre

Study coordinator:

Dr. CW Menke-van der Houven van Oordt, MD PhD
Department of Medical Oncology
VU University Medical Center
De Boelelaan 1117
1081 HV Amsterdam
Email: c.menke@vumc.nl
T: 020-444 4321 / F: 020-444 4079

Table of contents

1 Summary 5
2 Introduction 6
3 Objectives of the trial 9
3.1 Objectives 10
3.2 Endpoints 10
4 Patient selection criteria 10
4.1 Inclusion criteria 10
4.2 Exclusion criteria 10
5 Trial design 11
5.1 Participant recruitment 11
5.2 Pre-study evaluation and follow-up 11
5.3 Withdrawal of individual subjects 12
5.4 Treatment 12
5.4.1 Treatment with cetuximab 12
5.4.2 Standard toxicity management of cetuximab 12
5.5 Clinical endpoints 13
5.5.1 Response assessment 13
5.5.2 Immuno-PET acquisition 14
5.5.3 Data-analysis ^{89}Zr- cetuximab scans 14
5.5.4 [18F-]FDG-PET 14
5.5.5 Assessment skin toxicity 14
6 Investigational Medicinal Product 16
6.1 Production, procedures and analysis of ^{89}Zr-cetuximab 16
6.2 Drug accountability cetuximab 16
7 Statistical considerations 17
7.1 Evaluation - type of variable 17
7.2 Statistical design and analysis 17
7.3 Accrual and duration of study 18
7.4 Missing data 18
8 Translational research 19
8.1 Objectives of the translational research study 19
8.1.1. Pharmacokinetics/imaging kinetics of ^{89}Zr-cetuximab 19
8.1.2 To explore the potential association between EGFR saturation with cetuximab and treatment outcome 19
8.1.3 To investigate kinase activity in skin samples before and after treatment with cetuximab 19
8.2 List of the material to be obtained for each patient 19
8.3 Instructions for treating and storing samples 20
9 Safety reporting
10 Ethical considerations 22
11 Trial insurance 23
12 Administrative responsibilities 24
13 Trial sponsorship and financing 25
14 Publication policy 25
15 References 26

LIST OF ABBREVIATIONS AND RELEVANT DEFINITIONS

ABR	ABR form (General Assessment and Registration form) is the application form that is required for submission to the accredited Ethics Committee (ABR = Algemene Beoordeling en Registratie)
AE	Adverse Event
AR	Adverse Reaction
CA	Competent Authority
CCMO	Central Committee on Research Involving Human Subjects
CV	Curriculum Vitae
DSMB	Data Safety Monitoring Board
EU	European Union
EudraCT	European drug regulatory affairs Clinical Trials GCP Good Clinical Practice
IB	Investigator’s Brochure
IC	Informed Consent
IMP	Investigational Medicinal Product
IMPD	Investigational Medicinal Product Dossier
METC	Medical research ethics committee (MREC); in Dutch: medisch ethische toetsing commissie (METC)
(S)AE	Serious Adverse Event
SPC	Summary of Product Characteristics (in Dutch: officiële productinfomatie IB1-tekst)
Sponsor	The sponsor is the party that commissions the organization or performance of the research, for example a pharmaceutical company, academic hospital, scientific organization or investigator. A party that provides funding for a study but does not commission it is not regarded as the sponsor, but referred to as a subsidizing party.
SUSAR	Suspected Unexpected Serious Adverse Reaction
Wbp	Personal Data Protection Act (in Dutch: Wet Bescherming Persoonsgevens)
WMO	Medical Research Involving Human Subjects Act (Wet Medisch-wetenschappelijk Onderzoek met Mensen

1 Summary

Rationale and Objective:

3rd line standard treatment of patients with metastatic colorectal cancer (CRC) harboring K-ras wild type consists of anti-EGFR treatment with either cetuximab or panitumumab. This type of treatment has a modest but significant beneficial activity in this patient group with improved progression-free and overall survival. Although it is well known that patients with advanced CRC harboring a K-Ras mutation will not respond to anti-EGFR treatment, it is not understood why patients with K-Ras wild type CRC do not all benefit from this type of therapy. In order to optimize treatment of these patients as well as health care costs, it is extremely important to identify those patients who will respond to treatment with an EGFR inhibitor at an early stage.

We hypothesize that the differences in response to treatment with cetuximab are due to variability in the pharmacokinetics and -dynamics of the antibody. Thus, we hypothesize that patients who do not respond to anti-EGFR treatment, have insufficient drug levels in tumor tissue. We hypothesize that this is due to pharmacodynamic processes such as sequestration of cetuximab in the liver which expresses high levels of EGF receptor.

With the introduction of immuno-positron emission tomography (PET), an attractive novel option to visualize molecular interactions has been developed using the combination of PET with labeled mAbs. Cetuximab labeled with zirconium-89 (^{89}Zr) has been successfully generated (GMP) and is available for this study. Previous studies have shown excellent stability of this compound and ^{89}Zr is shown to be safe in humans. We will use ^{89}Zr-cetuximab to demonstrate tumor targeting by imaging and explore the relation of uptake with treatment response. With this approach we hope to achieve a better understanding of the mechanisms of action of this therapeutic mAb in metastasized CRC and eventually develop strategies that may improve efficacy of cetuximab treatment.

Study design: Single centre, two step non-randomized intervention study.

Study population: Patients with histopathologically confirmed advanced CRC with K-Ras wild type, aged ≥ 18 years, with a life expectancy of at least 12 weeks, who are candidates for anti-EGFR antibody monotherapy (3rd line palliative treatment).

Intervention: Patients will be treated with cetuximab. For pharmacodynamic purposes PET-imaging with ^{89}Zr-labelled cetuximab will be performed. In addition, two [18F-]-FDG PET-CT will be performed to explore early response. Patients will undergo blood sampling and two skin biopsies for pharmacodynamic purposes of ^{89}Zr-labelled cetuximab and kinase activity profiles, respectively.

Main study objective/endpoint: The main objective of the first part of the study is the demonstration of ^{89}Zr-cetuximab uptake in non-hepatic tumor lesions. The main objective of the second part is the association between ^{89}Zr-cetuximab uptake in non-hepatic tumor lesions and treatment outcome.

Nature and extent of the burden and risks associated with participation, benefit and group relatedness: Upon enrolment in this study, patients will be asked to undergo two skin biopsies during treatment. During therapy, follow-up will include standard laboratory analysis, immuno-PET-CT and [18F-]-FDG PET-CT on regular visits to the outpatient clinic. Side effects of the medication and adverse events as a consequence of the skin biopsies may occur. The radiation exposure is acceptable and requires no shielding after injection of ^{89}Zr-labelled cetuximab. Patients may benefit from disease regression or stabilization as cetuximab has proven clinical benefit in this patient population.

2 Introduction

2.1 Disease information

EGFR and colorectal cancer

Colorectal cancer (CRC) is the second cause of cancer death both in the United States and in Europe. In the Netherlands, around 10.000 new patients are diagnosed each year and around 5000 deaths occur annually (www.ikcnet.nl). Recently, therapeutic options have been expanded with the introduction of monoclonal antibodies against the epidermal growth factor receptor, i.e. cetuximab and panitumumab.

Epidermal growth factor receptor (EGFR), a member of the type I receptor tyrosine kinase family, is often upregulated in CRC and this has been associated with tumor aggressiveness and poor prognosis (Goldstein 2001). Activation of EGFR leads to activation of different downstream signaling pathways such as the Ras/Raf/MAP kinase pathway. This pathway plays a key role in the regulation of cell growth, differentiation, proliferation, apoptosis and cellular invasiveness. Activation of the PI3K/Akt pathway is regulated by EGFR as well as PTEN, deficiency of which has been associated with unsuppressed signaling activity (Heineman 2009 and ref therein).
Cetuximab is a chimeric mouse/human IgG1 monoclonal antibody targeted against the extracellular domain of the EGFR. Binding of cetuximab to the receptor prevents ligand binding, induces receptor internalization and causes a direct inhibition of the receptor tyrosine kinase activity (Mendelsohn 2003). Cetuximab may also induce antibody-dependent cell-mediated cytotoxicity (ADCC), but the clinical relevance of this effect is not clear. In several phase II and III clinical trials cetuximab has shown promising efficacy in patients with metastatic CRC, as single agent as well as in addition to chemotherapy (Cunningham 2004, Jonker 2007, van Cutsem 2009, Bokemeyer 2009). However, not all patients benefit. Mutations in downstream targets of EGFR (K-Ras/B-Raf) have been implicated in the resistance to anti-EGFR therapy, i.e. K-Ras mutations have been observed in 40% and B-Raf in 0-12.5% of CRC patients (reviewed in Heinemann 2009). Post hoc analyses are now available which evaluate the influence of K-Ras mutation status on treatment efficacy.
The CRYSTAL trial compared chemotherapy consisting of 5-fluorouracil, folinic acid and irinotecan (FOLFIRI) plus cetuximab to FOLFIRI alone as first-line chemotherapy in metastasized CRC patients. Retrospective analysis revealed that the addition of cetuximab to FOLFIRI caused a significant increase in overall response rate (ORR) in K-Ras wild type patients (43% vs. 59% p = 0.0025), while no improvement was observed in the mutant population (40 vs. 36% p=0.46) (van Cutsem 2009).
The OPUS trial investigated the 5-fluorouracil, folinic acid and oxaliplatin (FOLFOX) regimen with or without cetuximab in advanced CRC patients. As in the previous trial, cetuximab induced a significant increase in ORR in K-Ras wild type patients (37% vs. 61% p 0.011), whereas no effect on ORR was seen in K-Ras mutant patients (49% vs. 33% p 0.106) (Bokemeyer 2009).
For pre-treated patients, the literature contains many case-series and posthoc analysis of clinical trials with combining chemotherapy with cetuximab, which all point to the same consistent relation between K-Ras wild type status and response to anti-EGFR treatment in 10-40% of the patients (Benvenuti 2007, Lievre 2008, de Roock 2008). Posthoc analysis of monotherapy cetuximab shows a 12.8% response rate according to RECIST in K-Ras wild type patients (Karapatis 2008). The only prospective study of cetuximab as a single agent in pre-treated metastatic CRC patients showed that K-Ras wild type status was associated with a greater probability of disease control (p 0.0003) (Khambata-Ford 2007).

Dosage of cetuximab

A critical issue in clinical studies with an EGFR inhibitor is the definition of the optimal biologic dose and schedule. Based on pre-clinical studies, the optimal biological dose was defined as the dose at which EGFR was completely saturated, such that maximal inhibition of tumor growth would be

achieved. It was suggested that complete saturation of EGFR might be associated with a plateau of the systemic clearance of the antibody. Pharmacological analysis of different phase I studies resulted in the recommended loading and maintenance dose every week of 400 mg/m2 and 250 mg/m2, respectively (Baselga 2000, Robert 2001, Fracasso 2007). These doses are well tolerated and no DLTs have been observed up to 500 mg/m2 weekly. A more recent study showed a single DLT with 700 mg/m2 every two weeks (Tabernero 2009). Interestingly, the trough levels using the recommended dosage varied, and in some patients they were below those estimated to yield saturation (Robert 2001, Tan 2006). At this moment it remains unclear what if any relation exists between through levels and response.

Recently, biweekly in stead of weekly schedules of cetuximab administration, similar to the biweekly schedule of panitumumab, have been assessed in several studies. Biweekly schedules would allow greater flexibility to combine cetuximab with different chemotherapy regimes and result in a significantly decreased burden for patients. These studies show that a biweekly schedule with 500mg/m2 exhibits similar pharmacokinetics and pharmacodynamics as weekly dosing with 250 mg/m2 (Taberno 2009, Shitara 2010, Pfeiffer 2008, Martin-Martorel 2008). In addition, safety and efficacy are comparable, making biweekly administration of 500mg/m2 cetuximab an attractive and effective alternative to the standard weekly schedule.

As a class of targeted drugs, EGFR inhibitors do not show many of the severe side effects commonly observed with cytotoxic chemotherapy except for skin toxicity .Interestingly, a potential association between the severity of skin toxicity and a favorable response to anti-EGFR has been reported. Indeed, skin toxicity is believed to be an ‘on target’ effect (Cunningham 2004). In addition, hypomagnesia has been observed after treatment with cetuximab due to blockade of the EGF receptor in the kidney. Recently, it has been suggested that early magnesium reduction after treatment with cetuximab might serve as a marker for efficacy and outcome (Vincenzi 2008). Despite these observations, at this moment in time it is still unclear which patients may benefit from treatment with cetuximab and who will not, and ways to identify treatment failure in a timely fashion are urgently needed.

2.2 Immuno-PET

The introduction of immuno-positron emission tomography (PET), the combination of PET with mAbs, is an attractive novel option to visualize molecular interactions. In advanced CRC patients, treatment with cetuximab labeled with a positron emitter has the potential for quantification of the interactions of the drug within the different compartments of the body, including metastases. The positron emitters ^{89}Zr ($t_{1/2}$ = 78.4 h) is particularly well suited for imaging of slow kinetic intact antibodies because its long half-lives allows imaging at late time points for obtaining maximum information. VUmc has obtained a leading position in immuno-PET imaging by the introduction GMP (good manufacturing practice)-compliant procedures for the production and purification of positron emitters like ^{89}Zr, by the development of universal procedures for stable coupling of this positron emitters to MAbs, and by performing preclinical and clinical safety and proof of principle studies. There is no shielding required and the patient can go home directly. The first two days, the patient is not allowed in the proximity of children and pregnant women. Additional information regarding ^{89}Zr cetuximab can be found in SECTION 6.

2.3 Study rationale

Although it is well known that most patients with advanced CRC containing a K-Ras mutation will not respond to anti-EGFR treatment (Amado 2008, van Cutsem 2008, Bokemeyer 2008), it is unknown why not all patients with K-Ras wild type benefit from this type of therapy. Although mutations in other downstream signaling molecules (e.g. BRAF) may play a role, this does not explain it completely (di Nicolantonio 2008).

We hypothesize that differences in response to cetuximab treatment in patients with wild type K-Ras CRC are primarily due to variability in the pharmacokinetics and -dynamics of the antibody. Thus, we hypothesize that patients who do not respond to anti-EGFR treatment, have insufficient drug levels in tumor tissue. We hypothesize that this is due to pharmacodynamic processes such as sequestration of cetuximab in the liver which expresses high levels of EGF receptor.

Based on this hypothesis we have designed a two step exploratory pilot immuno-PET study for patients with metastasized, K-Ras wild type CRC who will be treated with cetuximab. In the first part, we will aim to demonstrate targeting of ^{89}Zr-cetuximab to non-hepatic tumor lesions. In vivo imaging in tumor-bearing mice has shown promising results regarding imaging the tumor lesions with ^{89}Zr-cetuximab (Aerts et al 2008). Previous studies have shown ^{89}Zr to be safe in humans (Borjesson 2006).

After having demonstrated uptake of ^{89}Zr-cetuximab, we will proceed to the second part of the study and assess the association between tumor uptake and response. Our aim will be to identify those patients that fail to show clinical benefit (i.e. complete response, partial response and stable disease according to RECIST 1.1 criteria). If this pilot study reveals that indeed cetuximab has an inter-patient differential pharmacokinetic profile with insufficient tumor homing associated with lack of response, we have planned a dose finding follow-up study using increasing cetuximab doses until tumor lesions are sufficiently targeted based on immuno-PET imaging.

3 Objectives of the trial

3.1 Objectives

3.1.1 Part one - Primary objective

3.1.2 To demonstrate uptake of ^{89}Zr-cetuximab in non-hepatic tumor lesions using immuno-PET when administered during the loading dose of cetuximab.

3.1.3 Part two - Primary objective

To investigate whether there is an association between uptake of cetuximab in non-hepatic tumor lesions and response according to RECIST 1.1 criteria.

3.1.4 Part two - Secondary objectives

1) To investigate whether there is an association between levels of uptake of ^{89}Zr-cetuximab in the liver compared to levels of uptake in non-hepatic tumor lesions.
2) To explore whether the response observed on [18F]-FDG-PET can serve as an early response marker for future response to targeted therapy according to RECIST 1.1.
3) To explore whether there is an association between ^{89}Zr-cetuximab uptake in non-hepatic tumor lesions, grade of skin toxicity and response according to RECIST 1.1.

3.2 Endpoints

3.2.1 Part one - Primary endpoint

The detection of ^{89}Zr-cetuximab uptake in non-hepatic tumor lesions (present/absent; present being defined as levels measured in ROI's > standard deviation of background +1).

3.2.2 Part two – primary endpoint

The % uptake (of total injected) ^{89}Zr-cetuximab in non-hepatic tumor lesions as measured in ROI's corrected for background levels.

3.2.3 Part two - secondary endpoints

1) The % uptake (of total injected) ^{89}Zr-cetuximab in liver lesions as measured in ROI's corrected for background levels.
2) [18F-]FDG PET measurements (SUV_{max}) before and after 4 weeks of treatment with cetuximab.
3) Grade of skin toxicity as measured by predefined criteria (see below).

3.2.1 Other study parameters

4) Serum magnesium levels before and during treatment.
5) EGFR saturation with cetuximab in skin samples.
6) Kinase activity in skin samples before and after treatment with cetuximab.
7) Pharmacokinetics of ^{89}Zr-cetuximab.

4 Patient selection criteria

4.1 Inclusion criteria

Subjects are eligible if they meet the following criteria:

- Advanced colorectal adenocarcinoma
- Subjects must have been treated according to standard care with a fluoropyrimidine (e.g. fluorouracil or capecitabine), irinotecan, and oxaliplatin or had contra-indications to treatment with these drugs.
- Age ≥ 18 years.
- Histological or cytological documentation of cancer is required.
- Tumor material must be tested wild type for the K-Ras gene.
- Subjects have at least one measurable lesion outside the liver. Lesions must be evaluated by CT-scan or MRI according to Response Evaluation Criteria in Solid Tumors (RECIST 1.1).
- ECOG Performance Status of 0, 1 or 2
- Adequate liver and renal functions as assessed by the following laboratory requirements to be conducted within 7 days prior to screening:
    - Total bilirubin ≤ 1.5 times the upper limit of normal
    - ALT and AST ≤ 2.5 times upper limit of normal (≤ 5 times upper limit of normal for subjects with liver involvement of their cancer)
    - Serum creatinin ≤ 1.5 times upper limit of normal or a calculated creatinin clearance ≥ 50 ml/min
- Signed informed consent must be obtained prior to any study specific procedures.

4.2 Exclusion criteria

Subjects who meet the following criteria at the time of screening will be excluded:

- Previous exposure to an anti-EGFR therapy
- Significant skin condition interfering with treatment
- Insulin dependency
- Pregnant or breast-feeding subjects. Women of childbearing potential must have a negative pregnancy test performed within 7 days of the start of treatment. Both men and women enrolled in this trial must agree to use adequate barrier birth control measures (e.g., cervical cap, condom, and diaphragm) during the course of the trial. Oral birth control methods alone will not be considered adequate on this study, because of the potential pharmacokinetic interaction between study drug and oral contraceptives. Concomitant use of oral and barrier contraceptives is advised. Contraception is necessary for at least 6 months after receiving study drug.
- Concurrent anticancer chemotherapy, immunotherapy or investigational drug therapy during the study or within 4 weeks of the start of study drug.
- Radiotherapy to the target lesions during study or within 4 weeks of the start of study drug. Palliative radiotherapy will be allowed.
- Major surgery within 28 days of start of study drug.
- Substance abuse, medical, psychological or social conditions that may interfere with the subject's participation in the study or evaluation of the study results.
- Any condition that is unstable or could jeopardize the safety of the subject and their compliance in the study.

5 Trial design

5.1 Participant recruitment

Patients will be included from the VUMc Medical Oncology Department and the Internal Medicine Department of the Medical Centre of Hoofddorp (Spaarne Ziekenhuis) in an outpatient setting. As on a monthly basis five of the aforementioned patients are diagnosed, approximately three patients per month are expected to be included. Therefore, accrual accomplishment is estimated within one year.

5.2 Pre-study evaluation and follow-up

Upon informed consent for participation in this trial, baseline evaluations except imaging are to be conducted within 14 days prior to start of protocol therapy. [18F]-FDG PET and CT and/or MRI will be done ≤ 4 weeks prior to start of therapy.

Five patients will be asked to undergo 3 additional PET scans (after the ^{89}Zr -cetuximab dose) and 3 simultaneous venous blood samples in order to study pharmacokinetics of ^{89}Zr -cetuximab in more detail.

Table 1 Standard evaluation, laboratory tests and follow-up

	Baseline	Start treatment	From week 5	End of study
**Week**	- 2 to 0	1,3 and 5	every 4 wks	
**History**	X	X	X	X
**Phys. exam**	X	X	X	X
**Skin biopsy**	X	After 3-4 wks of treatment		
**Toxicity**	X	X	X	X
**Chemistry [1]**	X	X	X	X
**Hematology[2]**	X	X	X	X
**Tumor marker (if applicable)**	X	every 4 wks	every 4 wks	X
**Tumor measurements[3]**	X		every 2 mths for the first year, every 3 mths after 1 year of treatment	X
**18FDG-PET[4]**	X		4 wks	
**Immuno PET-CT[5]**		0-2h, day 2, 3, 4 and 7<br>0-2h and day 4 or7		
**pharmacokinetics**		0-2h, day 2,3,4 and 7<br>1h and day 4 or 7		

[1]**Chemistry panel:** Na, K, Ca, Mg, Alb, creatinin, bilirubin, γGT, AF, ASAT, ALAT, LDH.

[2]**Hematology:** Hb, Ht, WBC, ANC, Platelets

[3]**Radiology:** MRI or CT images will be required within 4 weeks before start, every 2 months during the first year of treatment and thereafter every 3 months until progressive disease. CT scans or MRI scans will be performed according to standard procedures. The radiologist will review any responses using RECIST 1.1 independently from the investigator.

[4]**18FDG-PET:** scans will be required within 4 weeks before start and 4 weeks after start.

[5]**Immuno-PET-CT scan:** The first five patients will be asked to undergo in total 5 immuno-PET scans and 5 simultaneous venous blood samples at 0-2h, day 2,3,4 and 7 after administration of ^{89}Zr -cetuximab in order to study pharmacokinetics of ^{89}Zr -cetuximab in more detail. The following patients will undergo in only 2 immuno-PET scans, at 0-2h and 4 or 7 days after ^{89}Zr –cetuximab administration.

Follow up will last till progressive disease and/or death of the patient. In the case of progressive disease, the choice of any following line palliative chemotherapy is at the discretion of the investigator.

5.3 Withdrawal of individual subjects

A patient will be discontinued from the study therapy under the following circumstances:

-evidence of progressive disease
-upon patient's request for any reason
-unacceptable toxicity

Patients who withdraw their informed consent within the first 6 weeks while no medical need for withdrawal exists can be replaced at the discretion of the investigator's team. If during screening a patient's condition deteriorates and the patient becomes unfit for treatment, the patient will be replaced.

Patients withdrawn from treatment for any reason except progressive disease will continue follow up as specified above unless the patient declines.

5.4 Treatment

5.4.1 Treatment with cetuximab

Unlabelled cetuximab infusion

Unlabelled cetuximab will be given intravenously in a biweekly schedule with 500 mg per square meter of body surface area as initial and maintenance dose. An antihistamine will be given before each dose of cetuximab. Treatment will be continued until death, in the absence of the occurrence of unacceptable adverse events, tumor progression, worsening symptoms of the cancer, or request by the patient. The infusion will take place at the outpatient department of Medical Oncology of the VUmc. Afterwards, the patient is moved to the department of Nuclear Medicine & PET research of the VUmc where the infusion of the labeled part of the cetuximab will take place within two hours after administration of the unlabelled cetuximab.

^{89}Zr-abelled cetuximab infusion

For the labeled ^{89}Zr-cetuximab injection, the infusion line inserted for the unlabelled cetuximab will be used. ^{89}Zr-cetuximab will be injected as a bolus of 20mL followed by 10mL physiologic saline flushing. All relative times after treatment have to be understood with respect to the end of this infusion. This time will be considered as study time "0:00". (see SECTION 6 Investigational Medicinal product for details regarding ^{89}Zr –cetuximab).

5.4.2 Standard toxicity management of cetuximab

As a class of targeted drugs, EGFR inhibitors do not show many of the severe side effects commonly observed with cytotoxic chemotherapy except for skin toxicity. In most patients, this skin rash exhibit some degree of spontaneous partial improvement during therapy and it generally resolves completely without scaring following cessation of EGFR inhibition. In case of ≥ grade 3 toxicity follow table 2 for treatment adjustment.

Other side effects include hypersensitivity reactions. After appropriate measures have been taken treatment will be continued at a decreased infusion rate in case of a mild hypersensitivity reaction

(CTC 4.0 grade ≤2). In case of hypersensitivity reactions exceeding grade 2 treatment will be stopped (table 2).

Also reversible electrolyte deficiencies, dyspnoea, fatigue, asthenia and diarrhea have been reported, none exceeding grade 3 toxicity.

Table 2 Treatment adjustment to specific toxicities

**Toxicity**	**Severity**	**Dose adjustment/other measures**
Skin rash<br>Graded according to Table 3	Grade 1 and grade 2	No dose adjustment required.<br>Standard acne treatment can be started[1]
	Grade ≥3 as graded	Delay further treatment until skin rash has ameliorated to grade < 3[1],<br>Restart at maintenance dose
	Grade ≥3 persists > 3 weeks after dose delay of cetuximab	Subjects must be withdrawn from the treatment.
Hypersensitivity<br>Graded according to CTC4.0	Grade 1	Decrease the cetuximab infusion rate by 50% and monitor closely for any worsening.
	Grade 2	Stop cetuximab infusion.<br>Administer bronchodilators, oxygen, etc. as medically indicated.<br>Resume infusion at 50% of previous rate once allergic/hypersensitivity reaction has resolved or decreased to Grade 1 in severity, and monitor closely for any worsening
	Grade 3 or grade 4	Stop the cetuximab infusion immediately and disconnect infusion tubing from the subject.<br>Administer epinephrine, broncho- dilators, antihistamines, glucocorticoids, intravenous fluids, vasopressor agents, oxygen, etc., as medically indicated.<br>Subjects must be withdrawn immediately from the treatment and must not receive any further cetuximab treatment.

[1]Standard acne treatment is allowed and may provide some benefit. Treatment according to the Nederlands Huisartsen Genootschap will be allowed using topical applications of benzoylperoxide hydrogel 5% (50 mg/g) once daily or tretinoïne (Vitamine A acid) crème 0.05% (0.5 mg/g), once daily in the evening. In the absence of improvement, clindamycine hydrochloride lotion 1% (10 mg/ml), once daily in the morning may be applied. Ultimately, switching to doxycycline while continuing tretinoïne or benzoylperoxide therapy is allowed but consultation of a dermatologist is strongly recommended in this case.

[2]In case of severe skin toxicity (grade ≥3) treatment will be withheld and dermatologic therapy will be started (standard acne treatment, see above). Once skin toxicity improves (≤ grade 2), cetuximab will be restarted at maintenance dosage level. In case of persistent severe skin toxicity, cetuximab will be discontinued**.**

5.5 Clinical endpoints

5.5.1 Response assessment

Response will be assessed clinically and radiological (CT scan or MRI). The RECIST (version 1.1) criteria for response will be used, encompassing the categories complete response, partial response, stable disease progressive disease and inevaluable for response (Eisenhauer 2009).
In addition, individual tumor measurements will be collected in order to calculate % change in axis length (long axis for metastatic lesions; short axis for lymph nodes). All tumor assessments are conducted by the investigator, and all responses are independently reviewed by a radiologist.

5.5.2 Immuno-PET acquisition

All PET studies will be performed on a Gemini TF-64 PET-CT scanner. In 5 patients, whole-body PET scans (trochanter major femora-skull vertex) will be acquired after a single injection of 37 MBq ^{89}Zr- cetuximab on day 1 (0-2h after injection), day 2, 3, 4 and 7 for pharmacokinetic purposes. In the remaining patients, PET scans will be acquired on day 1 and day 4 or 7 depending on the optimal time point determined with the results of the first five patients. The venous infusion line inserted for administration of unlabelled cetuximab will be used for ^{89}Zr- cetuximab injection as well. At each scan a second venous cannula will be inserted for blood sampling. Blood samples will be used to measure absolute radioactivity concentration in whole blood and plasma. Prior to each whole-body PET scan, a 30 mAs low-dose CT scan will be acquired for attenuation correction and localization purposes. PET scans will consist of 10-12 bed positions, depending on the length of the patient, of 5 min each. Total acquisition time per scan, including LD-CT, will be below 90 min.

5.5.3 Data-analysis ^{89}Zr- cetuximab scans

All PET data will be corrected for dead time, scatter, randoms, decay, tissue attenuation. PET/CT data will be reconstructed with TF-OSEM, resulting in a transaxial spatial resolution of ~7 mm in the centre of the field of view. Regions of interest (ROIs) will be defined using the CT images which are co-registered to the PET/CT data. ROIs around the organs which accumulate ^{89}Zr (e.g. liver, lung, and metastatic lesions) will be drawn manually directly on the PET images. All tissue concentrations will be related to the blood concentration. The radioactivity concentrations in all regions for all imaging time points should be recorded digitally in a spread sheet. A tumor lesion will be scored positive if there will be uptake above background, being defined as SD(background) +1.
For pharmacokinetics, absorbed dose calculations will be made using the Olinda/EXM 1.0 software package, based on the Medical Internal Radiation Dose scheme (Stabin 2005).

5.5.4 [18F-]FDG-PET

To allow for flexible logistics, these studies will be conducted using either PET-CT or PET, using the same scanner for each individual. Studies and scanner calibrations will fully comply with the EANM guidelines (EJNMMI 2010, Boellaard 2010). The dose of [18F]-FDG will be about 185 MBq for PET-CT. Scan trajectory (WB-mode) will be adapted according to metastatic pattern in individual patients, and will remain the same after the baseline scan. Data-analysis will be done using in-house developed software, yielding SUV max, 70%VOI, 50%VOI, normalized for serum glucose, body weight, LBM and BSA.

5.5.5 Assessment skin toxicity

Cutaneous adverse effects due to EGFR inhibition are most commonly a papulopustular reaction involving most frequently areas of the head and upper trunk. Changes of the hair and nails may also occur. The CTC 4.0 criteria appear to be insufficiently specific to monitor these changes, as they rely heavily on body surface area as a determinant for rash severity. Thus, they fail to account for the location, i.e. upper trunk and face, and the subjective discomfort of EGFR inhibitor mediated rash. An EGFR inhibitor specific grading system has been developed which reflects the specific nature of an EGFR inhibitor-mediated skin toxicity (Melosky 2009, see table 3 below).

Table 3 Grading system for skin rash mediated by EGFR inhibition

Grade	Skin phenotype	Symptoms	Effect on daily life	Rating
Grade 1	macular or papular eruptions or erythema	no associated symptoms		mild
Grade 2A		pruritus or other symptoms	symptomatic but tolerable	moderate
Grade 2B			symptomatic interfering with daily life	
Grade 3	severe, generalized erythroderma or macular, papular or vesicular eruptions			severe
Grade 4	generalized exfoliative, ulcerative or blistering skin toxicity			

6 Investigational Medicinal Product

6.1 Production, procedures and analysis of ^{89}Zr-cetuximab

Using standard procedures for the production of ^{89}Zr-mAb conjugates, several clinical ^{89}Zr-immuno-PET studies have been performed, are ongoing, or will be started shortly. In these ongoing studies safety was confirmed. ^{89}Zr is coupled to cetuximab via the bifunctional chelate desferal. ^{89}Zr will be produced at the VUmc campus by Cyclotron BV according to procedures essentially as described before (Verel 2003). Radiochemical purity of the purified ^{89}Zr used for labeling will be according to the specification as described in same paper. ^{89}Zr-MAb conjugates produced in this way were found to be fully stable in vitro, in tumor-bearing nude mice (Perk 2005, Aerts 2009), as well as in patients (van Dongen pers. comm.). Using the same procedures, the following conjugates have been produced and administered to patients without any adverse event: ^{89}Zr-cMAb U36 (Borjesson 2006), ^{89}Zr-ibritumomab tiuxetan (zevalin™) (Perk 2006), ^{89}Zr-rituximab (ritixan™), and ^{89}Zr-trastuzumab (herceptin™).

Radiolabeling of cetuximab with ^{89}Zr will be performed by the department of Otolaryngology/Head & Neck Surgery of the VU University Medical Centre according to Standard Operating Procedures (SOPs) within the premises of the Radionuclide Centre (RNC) of the Department of Nuclear Medicine & PET research. The whole process will be performed according to state-of-the-art Good Manufacturing Practice (GMP) standards. This production site was recently inspected by the Dutch Health Care Inspectorate and is licensed to manufacture tracers for human use according to the latest EU guidelines. This site has also been audited by the largest EU pharma companies.

The procedures for radiolabeling of cetuximab with ^{89}Zr have been validated with respect to the final quality of the prepared conjugate. In three independent labeling experiments, the percentage of label bound to the antibody as assessed by ITLC, HPLC and SDS-PAGE should always be > 90%, preferably > 95%, while the immunoreactive fraction as assessed by cell binding assay, should always exceed 50%, preferably 70%. This analysis will be performed according to a procedure essentially as described by Lindmo et al (Lindmo 1984). In this clinical investigation the radiochemical purity of each ^{89}Zr-labeled antibody batch prepared will be assessed by ITLC or HPLC and should be more than 90% to allow administration to a patient.

Sterility of each ^{89}Zr-labeled cetuximab batch will be assured by performing a media fill immediately after final filter sterilization of each batch. In addition, pyrogen testing will be performed according to European Pharmacope. In general, the expiration time of conjugates is 36 hours.

Radiation exposure

For the patient the total dose of Zirconium-89 is 37 MBq. After injection no shielding is required, and the patient can go directly home. There are no limitations to the adult residence of the patients. However the first 2 days the patient is not allowed in the proximity of children and pregnant women, and the first two weeks restricted to spend more than 4 hours a day in the same room with children and pregnant women. Also the first week it is advised to retain distance to children and pregnant women.

6.2 Drug accountability cetuximab

Unlabelled cetuximab will be obtained from the hospital pharmacist. Cetuximab is supplied as standard and will be prescribed in the outpatient clinic of the department of Medical Oncology at the VUMc. With regard to the ^{89}Zr-labelled cetuximab, thirty-six hours may elapse between radiolabeling of the compound and administration to the patient.

7 Statistical considerations

7.1 Evaluation - type of variable

1. Uptake of ^{89}Zr-cetuximab in non-hepatic tumor lesions (present/absent)
2. Uptake of ^{89}Zr-cetuximab in non-hepatic tumor lesions (% of total dose injected) (continuous)
3. Uptake of ^{89}Zr-cetuximab in liver (% of total dose injected)
4. Response according to RECIST 1.1 (categories)
5. Response according to% change of non-hepatic tumor lesion long axis / lymph node short axis
6. [18F]-FDG SUV_{max}
7. Grade skin toxicity
8. Serum magnesium level

7.2 Statistical design and analysis

7.2.1 Part one

Primary endpoint: maximum 11 patients + 1 allowing for drop-outs =12

Statistical design: probability

We will analyze ^{89}Zr-cetuximab uptake in non-hepatic tumor lesions as binary variable (present/absent). We assume that all patients with response (i.e. complete response and partial response) according to RECIST 1.1 criteria and half the patients with stable disease according to RECIST 1.1 criteria will show uptake. According to previous publications we estimate that ≥ 10% of patients will show response and 30% have stable disease. Thus assuming that the proportion of patients with uptake is 0.25 or more, the probability of seeing no uptake in a cohort of 11 patients is less the 0.05. This has been calculated using a binominal distribution (n=11, p=0.25).

Stopping rule: The trial will stop if 11 patients do not show uptake. The trial will proceed to part two when the first patient with uptake is seen.

7.2.2 Part two

Primary endpoint: 35 patients (including all patients of part one) + 3 allowing for drop-outs = 38

Statistical design: Comparison of two independent groups of binary data

We are planning a study of independent cases (= all patients with complete response, partial response and stable disease according to RECIST 1.1 criteria) and controls (= patients with progressive disease according to RECIST 1.1 criteria) with 1.5 control(s) per case. We assume a false positive rate of 0.2 in the true negative's (= patients with progressive disease according to RECIST 1.1 criteria) because other downstream targets of the EGFR/RAS pathway may be mutated, thus interfering with response to cetuximab treatment. Measurement of cetuximab uptake using ^{89}Zr-cetuximab will only be clinically relevant if we identify ≥ 70% of patients correctly as failures to treatment. Type I error probability has been set at 0.1, the power at 0.9. We will use a continuity-corrected chi-squared statistic or Fisher's exact test to evaluate this null hypothesis.

Secondary endpoints:

1. The explained variation of % change in tumor axis (short axis for lymph nodes, long axis for non-hepatic metastatic lesions) explained by % (of total injected) ^{89}Zr-cetuximab uptake in the non-hepatic tumor lesions will be analyzed. Depending on the data we will estimate this relationship fitting either a linear or non-linear regression curve.

2. The potential association between % of total injected ^{89}Zr-cetuximab uptake in the liver and % uptake in the non-hepatic tumor lesions will be analyzed by linear regression models.

3. The potential association of the change in [18F]-FDG SUV_{max} and response according to RECIST 1.1 will be analyzed with a comparison of the mean using ANOVA or Kruskall-Walls depending on the distribution.

4. The potential association between grade of skin toxicity and % (of total injected) ^{89}Zr-cetuximab uptake in the non-hepatic tumor lesions will be analyzed with a comparison of the mean using ANOVA or Kruskall-Walls depending on the distribution.

7.3 Accrual and duration of study

Estimated accrual for this study is 3 patients a month. Thus, patient accrual is expected to be completed within 12 – 14 months. Additional time is required to allow the response data to mature. Expected duration of follow-up after the end of accrual is 12 months (no maximum).

7.4 Missing data

All of the patients registered in the study will be accounted for. The number of patients who were not evaluable, who died or withdrew during follow up will be specified and censored. The distribution of follow up time will be described and the number of patients lost to follow-up will be given.

8 Translational research

8.1 Objectives of the translational research study

8.1.1 Pharmacokinetics/imaging kinetics of ^{89}Zr-cetuximab

Five patients will have in total 5 immuno-PET scans at 1h, day 2, 3, 4 and 7 after administration of ^{89}Zr-cetuximab with concomitant blood samples at each scan time point to do the pharmacokinetics and imaging kinetics of ^{89}Zr-cetuximab.

8.1.2 To explore the potential association between EGFR saturation with cetuximab and treatment outcome

EGFR is highly expressed in epidermal tissue, and thus is expected to bind a large amount of cetuximab. We are interested to explore the level of saturation with the hypothesis that a high level of saturation may well be associated with a high uptake in tumor lesions and with response to treatment. If future trials would confirm such a relationship, saturation of epidermal EGFR with cetuximab may be used as predictive marker for treatment response to cetuximab.

An immunohistochemistry assay using cetuximab on snap-frozen tissue biopsies has been developed by Shin et al to investigate this (Shin 2001). We have experience with this assay at our laboratory. We will perform this assay on the snap-frozen skinbiopsies.

8.1.3 To investigate kinase activity in skin samples before and after treatment with cetuximab

Microarrays of kinase substrate peptides have been developed to study cellular signal transduction by detecting up and downregulation of kinase activity in an array based format (such as PamChip® array, validated by Sikkema et al 2009). By generating information on kinase-induced peptide phosphorylation, these arrays may provide new insights into specific downstream signaling pathways. Ideally, kinase peptide substrate arrays provide kinase activity values across the kinome.

Kinase peptide substrate arrays consist of immobilized peptides containing Ser, Thr or Tyr residues with additional sequence context for phosphorylation by their upstream kinases and can therefore reveal kinase activity *in-vitro*. After incubating the array with cell lysate, phosphorylation is determined by fluorescence microscopy (anti-phospho antibodies). Spot intensities can subsequently be correlated with kinase activity (Piersma, Labots et al pers comm).

By analyzing skin biopsies, which express significant amounts of the EGFR receptor, we hope to identify changes in kinase activity before and after treatment with cetuximab, which potentially improves our understanding of the mechanism of action of this antibody and as such can assist in improving anti EGFR-therapy.

8.2 List of the material to be obtained for each patient

8.2.1 Blood samples

From 5 patients blood samples (20 ml) will be collected at 0-2h, on day 2, 3, 4 and 7, from the remaining patients a blood sample will be collected at 0-2h and on day 4 or 7 for pharmacokinetic purposes.

.

8.2.2 Skin biopsies

Optional (separate consent form)

If a patient has agreed to have a skin biopsy, a punch biopsy of the skin will be performed within 14 days before start of cetuximab treatment and after 3-4 weeks of treatment.

8.3 Instructions for treating and storing samples

8.3.1 Blood samples

The bloodsamples will be collected and stored according to local practice in our reasearch facility.

8.3.2 Skin tissue specimen

Immediately after skin biopsy, tissue will be snap frozen in liquid nitrogen and stored at -80 °C in our research facility. Next, cryoslides of frozen tissue will be made for the lysis procedure as well as for H&E staining and immunohistochemistry analysis.

The lysis procedure includes addition of a sample buffer with protease inhibitors and phosphatase inhibitors to a cryoslides containing vial. After centrifugation the supernatant will be aliquotted and stored at –80. A small sample (1 or 2 µl) will be used for protein quantification. The lysate will be used for the PamChip®.

The immunohistochemistry analysis will be performed as described by Shin et al (Shin 2001).

9 Safety reporting

9.1 Section 10 WMO event

In accordance to section 10, subsection 1, of the WMO, the investigator will inform the subjects and the reviewing accredited METC if anything occurs, on the basis of which it appears that the disadvantages of participation may be significantly greater than was foreseen in the research proposal. The study will be suspended pending further review by the accredited METC, except insofar as suspension would jeopardize the subjects' health. The investigator will take care that all subjects are kept informed.

9.2 Adverse and serious adverse events

Adverse events are defined as any undesirable experience occurring to a subject during a clinical trial, whether or not considered related to the investigational drug. All adverse events reported spontaneously by the patient or observed by the investigator or his staff will be recorded.

A serious adverse event is any untoward medical occurrence or effect that at any dose results in death;

- is life threatening (at the time of the event);
- requires hospitalization or prolongation of existing inpatients' hospitalization;
- results in persistent or significant disability or incapacity;
- is a congenital anomaly or birth defect;

All SAEs will be reported to the accredited METC that approved the protocol, according to the requirements of that METC.

9.3 Annual safety report

The investigators will submit, once a year throughout the clinical trial, a safety report to the accredited METC.

9.4 Follow-up of adverse events

All adverse events will be followed until they have abated, or until a stable situation has been reached. Depending on the event, follow up may require additional tests or medical procedures as indicated, and/or referral to the general physician or a medical specialist.

10 Ethical considerations

10.1 Patient protection

The study will be conducted according to the principles of the declaration of Helsinki and in accordance with the Medical Research Involving Human Subjects Act (WMO).

Subjects will be identified in the trial database by unique anonymous identification numbers.

10.2 Informed consent

The informed consent document will be used to explain the risks and benefits of study participation to the patient in simple terms before the patient is entered into the study. It will be emphasized that participation is voluntarily and consent can be retracted at any time.

The investigator is responsible to see that informed consent is obtained from each patient and to obtain the appropriate signatures and dates on the informed consent document prior to the performance of any protocol procedures and prior to the administration of study drug.

10.3 Benefits and risks assessment

10.3.1 Risks

Use of positron emitting radionuclides means exposure to ionizing radiation. The total effective dose of one administration of Zirconium-89 MAb will be about 20 mSv. Low-dose CT will deliver 3 mSv per CT. [18F-]FDG yields a radiation dose of 3.3 mSv for 185MBq.

When considering the justification of application, a risk to benefit analysis has to be performed. ICRP-62 provides a model for this. According to this model, the risk level corresponds to “moderate”, while the social benefit corresponds to “substantial”.

After injection of the conjugate no shielding is required, and the patient can go directly home without further limitations. The risks of cetuximab are well-known as this drug is used in routine clinical practice (see above for detailed description). The first 6 months after the study both males and females are advised to prevent pregnancy.

10.3.2 Potential benefits

Cetuximab has been approved as 3rd line treatment for advanced CRC with K-Ras wild type status. Results from recent phase II and III trials have shown that cetuximab is an effective treatment for these patients.

11 Trial insurance

The sponsor/investigator has a liability insurance which is in accordance with article 7, subsection 6 of the WMO.

The sponsor (also) has an insurance which is in accordance with the legal requirements in the Netherlands (Article 7 WMO and the Measure regarding Compulsory Insurance for Clinical Research in Humans of 23th June 2003). This insurance provides cover for damage to research subjects through injury or death caused by the study.

1. € 450.000,-- (i.e. four hundred and fifty thousand Euro) for death or injury for each subject who participates in the Research;
2. € 3.500.000,-- (i.e. three million five hundred thousand Euro) for death or injury for all subjects who participate in the Research;
3. € 5.000.000,-- (i.e. five million Euro) for the total damage incurred by the organization for all damage disclosed by scientific research for the Sponsor as 'verrichter' in the meaning of said Act in each year of insurance coverage.

The insurance applies to the damage that becomes apparent during the study or within 4 years after the end of the study.

12 Administrative responsibilities

Principal investigator
Prof. dr. H.M.W. Verheul, MD PhD
Head Department of Medical Oncology
VU University Medical Center
De Boelelaan 1117
1081 HV Amsterdam
E: h.verheul@vumc.nl
T: 020-444 4321 / F: 020-444 4079

Trial-coordinator
Dr. CW Menke-van der Houven van Oordt, MD PhD
Department of Medical Oncology
VU University Medical Centre
De Boelelaan 1117
1081 HV Amsterdam
E: c.menke@vumc.nl / T: 0031-20-4442130

Subinvestigators
Prof Dr. GAMS van Dongen, PhD
Department of Otolaryngology/Head and Neck Surgery
VU University Medical Centre

Prof Dr. OS Hoekstra, MD PhD
Dr Ir A van Lingen, PhD
Dr Ir MC Huisman, PhD
Dr. D Vugts, PhD
Department Nucl Med & PET research
VU University Medical Centre

Dr. JJ van der Vliet, MD PhD
M Labots MD
Department of Medical Oncology
VU University Medical Centre

C van Montfrans, MD
Department of Dermatology
VU University Medical Centre

A Beeker, MD
Department of Internal Medicine
Medical Centre Hoofddorp (Spaarne Ziekenhuis)
Spaarnepoort 1
2134 TM Hoofddorp

Data management

CRFs for the recording of all data will be developed by the data manager of the VUMC. The forms will be printed on normal paper and the original CRFs will be retained by the investigator. Data should be recorded legibly onto the CRF in black, permanent ink, ballpoint pen. Corrections should be made legibly and initially and dated by approved personnel, the reasons for significant changes must be provided. Correction fluid or covering labels must not be used.
CT and MRI data will be stored at the IMS-server for at least two years after the last patient of the study has been evaluated or longer if necessary.
Immuno-PET-CT data will be stored in regular diagnostic databases of the nuclear medicine department.

Amendments
Amendments are changes made to the research after a favorable opinion by the accredited METC has been given. All amendments will be notified to the METC that gave a favorable opinion.

A ‘substantial amendment’ is defined as an amendment to the terms of the METC application, or to the protocol or any other supporting documentation, that is likely to affect to a significant degree:

- the safety or physical or mental integrity of the subjects of the trial;
- the scientific value of the trial;
- the conduct or management of the trial; or
- the quality or safety of any intervention used in the trial.

All substantial amendments will be notified to the METC and to the competent authority.

Non-substantial amendments will not be notified to the accredited METC and the competent authority, but will be recorded and filed by the sponsor.

Annual progress report
The investigator will submit a summary of the progress of the trial to the accredited METC once a year. Information will be provided on the date of inclusion of the first subject, numbers of subjects included and numbers of subjects that have completed the trial, serious adverse events/ serious adverse reactions, other problems, and amendments.

End of study report
The investigator will notify the accredited METC of the end of the study within a period of 8 weeks. The end of the study is defined as the last patient’s last visit.

In case the study is ended prematurely, the investigator will notify the accredited METC, including the reasons for the premature termination.

Within one year after the end of the study, the investigator/sponsor will submit a final study report with the results of the study, including any publications/abstracts of the study, to the accredited METC.

13 Trial sponsorship and financing

Investigator-initiated study.

Financing: Department of Medical Oncology, Department of Nuclear Medicine and PET research, Department of Otolaryngology/Head and Neck Surgery; VU University Medical Centre.

14 Publication policy

With the exception of personal and confidential medical records, all data generated under the trial shall be the property of the investigator. The investigator will publish or present the data generated in the study in accordance with accepted scientific practice.
The trial-coordinator and the principal investigator together with the co-investigators will prepare the manuscript reporting the final results.

15 References

Goldstein NS *et al*. Epidermal growth factor receptor immunohistochemical reactivity in patients with American Joint Committee on Cancer Stage IV colon adenocarcinoma: implications for a standardized scoring system. *Cancer* 2001; **92.**: 1331-46

Heinemann *et al.* Clinical relevance of EGFR- and KRAS-status in colorectal cancer patients treated with monoclonal antibodies directed against EGFR. *Canc Treat Rev* 2009; **35**: 262-71.

Mendelsohn J and Baselga J. Status of epidermal growth factor receptor antagonists in the biology and treatment of cancer. *J Clin Oncol* 2003; **21**:2878-99.

Cunningham D *et al.* Cetuximab monotherapy and cetuximab plus irinotecan in irinotecan-refractory metastatic colorectal cancer. *N Eng J Med* 2004; **351**: 337-45.

Jonker DJ *et al.* Cetuximab for the treatment of colorectal cancer. *N Eng J Med* 2007; **357**: 2040-8.

Van Cutsem E *et al.* Cetuximab and chemotherapy as initial treatment for metastatic colorectal cancer. *N Eng J Med*; 2009: **360**; 1408-17.

Bokemeyer C *et al.* Fluorouracil, leukovorin, and oxaliplatin with and without cetuximab in the first-line treatment of metastatic colorectal cancer. *J Clin Oncol*; 2009; **27**:663-71.

Benvenuti S et al Oncogenic activation of the RAS/RAF signaling pathway impairs the response of metastatic colorectal cancers to anti-epidermal growth factor receptor antibody therapies. Cancer Res 2007; **67**: 2643-8.

Lievre A. *et al. KRAS* mutations as an independent prognostic factor in patients with advanced colorectal cancer treated with cetuximab. *J Clin Oncol.* 2008; **26**:374-9.

De Roock, W. *et al.* KRAS wild-type state predicts survival and is associated to early radiological response in metastatic colorectal cancer treated with cetuximab. *Ann Oncol* 2008; **19**: 508-15.

Karapatis CS *et al* K-ras mutations and benefit from cetuximab in advanced colorectal cancer. *N Engl J Med*. 2008; **359**:1757-65.

Khambata-Ford S *et al* Expression of epiregulin and amphiregulin and K-ras mutation status predict disease control in metastatic colorectal cancer patients treated with cetuximab. *J Clin Oncol*. 2007;**25**:3230-7

Baselga J. Phase I studies of anti-epidermal growth factor receptor chimeric antibody C225 alone and in combination with cisplatin. *J Clin Oncol* 2000; **18**:904-14

Robert F. Phase I study of anti-epidermal growth factor receptor antibody cetuximab in combination with radiation therapy in patients with advanced head and neck cancer. *J Clin Oncol.* 2001; **19**:3234-43.

Fracasso PM. A phase I escalating single-dose and weekly fixed dose study of cetuximab: pharmacokinetic and pharmacodynamic rationale for dosing. *Clin Cancer Res* 2007; **13**:986-93.

Tabernero J et al. Cetuximab administered once every second week to patients with metastatic colorectal cancer: a two-part pharmacokinetic/pharmacodynamic phase I dose-escalation study. *Ann Oncol* 2009; 21:1537-45.

Pfeiffer P et al Biweekly cetuximab and irinotecan as third-line therapy in patients with advanced colorectal cancer after failure to irinotecan, oxaliplatin and 5-fluorouracil. *Ann Oncol 2008* 19:1141-1145.

Shitara K et al. Phase II study of combination chemotherapy with biweekly cetuximab and irinotecan for wild type KRAS metastatic colorectal cancer refractory to irinotecan, oxaliplatin and fluoropyrimidines. *Invest New Drugs* 2010 Epub ahead of print

Martin –Martorell P et al. Biweekly cetuximab and irinotecan in advanced colorectal cancer patients progressing after at least one previous line of chemotherapy: results of a phase II single institution trial. *Br J of Canc* 99:455-458.

Tan AR. Pharmacokinetics of cetuximab after administration of escalating single dosing and weekly fixed dosing in patients with solid tumours. *Clin Cancer Res* 2006; **12**:6517-22

Vincenzi B*et al* Early magnesium reduction in advanced colorectal cancer patients treated with cetuximab plus irinotecan as predictive factor of efficacy and outcome. Clin Canc Res 2008; **14**:4219-24.

Amado RG *et al.* Wild type *KRAS* is required for panitumumab efficacy in patients with metastatic colorectal cancer. *J Clin Oncol* 2008; **26**: 1626-34.

Van Cutsem E *et al* An open-label, single-arm study assessing safety and efficacy of panitumumab in patients with metastatic colorectal cancer refractory to standard chemotherapy. *Ann Oncol* 2008; **19**: 92-8.

Di Nicolantonio F *et al.* Wild type BRAF is required for response to panitumumab or cetuximab in metastatic colorectal cancer. J *Clin Oncol* 2008; **26**: 5705-12.

Aerts HJ et al. Disparity between in vivo EGFR expression and 89Zr-labeled cetuximab uptake assessed with PET. J Nucl Med 2009 **50**:123-151.

Borjesson PK *et al* Performance of immune-positron emission tomography with zirconium-89-labeled chimeric monoclonal antibody U36 in the detection of lymph node metastasis in head and neck cancer patients. *ClinCanc Res***12**:2133-40.

Eisenhauer EA et al. New response evaluation criteria in solid tumours: Revised RECIST guideline (version 1.1). Eur J of Canc 2009; **45**:228-47.

Stabin MG, OLINDA/EXM: the second-generation personal computer software for internal dose assessment in nuclear medicine. *J Nucl M* 2005; **46**:1023-7.

Boellaard R *et al* FDG PET and PET/CT: EANM procedure guidelines for tumor PET imaging; Version 1.0. *Eur J Nucl Med Mol Imaging* 2010; **37**:181-200.

Melosky B. Management of skin rash during EGFR-targeted monoclonal antibody treatment of gastrointestinal malignancies: Canadian recommendations. *Curr Oncol.*2009; **16**:16-26.

Verel I *et al.* 89Zr immuno-PET: comprehensive procedures for the production of 89Zr labeled monoclonal antibodies. *J Nucl Med* 2003 **44**: *1271-81.*

Perk LR et al. 89Zr as a PET surrogate radioisotope for scouting biodistribution of the therapeutic radiometals 90Y and 177Lu in tumor-bearing nude mice after coupling to the internalizing antibody cetuximab. J Nucl Med 2005 **46**:1898-1906.

Perk LR *et al* Preparation and evaluation of (89)Zr-Zevalin for monitoring of (90)Y-Zevalin biodistribution with positron emission tomography. *Eur J Nucl Med Mol Imag* 2006; **33**:1337-45.

Lindmo *et al*. Determination of the immunoreactive fraction of radiolabeled monoclonal antibodies by linear extrapolation to binding at infinite antigen excess. *J Immunol Meth*1984 **72**:77-89

Sikkema AH *et al*. Kinome profiling in pediatric brain tumors as a new approach for target discovery. *Cancer Res.* 2009; **69**:5987-95.

Shin DM *et al* Epidermal growth factor receptor-targeted therapy with C225 and cisplatin in patients with head and neck cancer. *Clin Canc Res* 2001; **7**:1204-13.
