# Supplementary material for: Early 18F-FDG PET/CT Evaluation Shows Heterogeneous Metabolic Responses to Anti-EGFR Therapy in Patients with Metastatic Colorectal Cancer
Source: PLoS One. 2016 May 19;11(5):e0155178. doi: 10.1371/journal.pone.0155178 (PMC4873260; doi:10.1371/journal.pone.0155178)
Supplement: S1 Table — (DOCX) [file pone.0155178.s006.docx]

| **S1 Table. In- and Exclusion Criteria** |  |
| --- | --- |
| **Inclusion criteria** | **Exclusion criteria** |
| 1. Advanced colorectal adenocarcinoma 2. Subjects must have been treated according to standard care with a fluoropyrimidine (e.g. fluorouracil or capecitabine), irinotecan, and oxaliplatin or had contra-indications to treatment with these drugs. 3. Age ≥ 18 years. 4. Histological or cytological documentation of cancer is required. 5. Tumor material must be tested wild type for the K-Ras gene. 6. Subjects have at least one measurable lesion outside the liver. Lesions must be evaluated by CT-scan or MRI according to Response Evaluation Criteria in Solid Tumors (RECIST 1.1). 7. ECOG Performance Status of 0, 1 or 2 8. Adequate liver and renal functions as assessed by the following laboratory requirements to be conducted within 7 days prior to screening:  - Total bilirubin ≤ 1.5 times the upper limit of normal - ALT and AST ≤ 2.5 times upper limit of normal (≤ 5 times upper limit of normal for subjects with liver involvement of their cancer) - Serum creatinin ≤ 1.5 times upper limit of normal or a calculated creatinin clearance ≥ 50 ml/min - Signed informed consent must be obtained prior to any study specific procedures. | 1. Previous exposure to an anti-EGFR therapy 2. Pregnant or breast-feeding subjects. Women of childbearing potential must have a negative pregnancy test performed within 7 days of the start of treatment. Both men and women enrolled in this trial must agree to use adequate barrier birth control measures (e.g., cervical cap, condom, and diaphragm) during the course of the trial. Oral birth control methods alone will not be considered adequate on this study, because of the potential pharmacokinetic interaction between study drug and oral contraceptives. Concomitant use of oral and barrier contraceptives is advised. Contraception is necessary for at least 6 months after receiving study drug. 3. Insulin dependency 4. Concurrent anticancer chemotherapy, immunotherapy or investigational drug therapy during the study or within 4 weeks of the start of study drug. 5. Major surgery within 28 days of start of study drug. 6. Substance abuse, medical, psychological or social conditions that may interfere with the subject’s participation in the study or evaluation of the study results. 7. Radiotherapy to the target lesions during study or within 4 weeks of the start of study drug. Palliative radiotherapy will be allowed. 8. Any condition that is unstable or could jeopardize the safety of the subject and their compliance in the study. |
