# Supplementary material for: Early 18F-FDG PET/CT Evaluation Shows Heterogeneous Metabolic Responses to Anti-EGFR Therapy in Patients with Metastatic Colorectal Cancer
Source: PLoS One. 2016 May 19;11(5):e0155178. doi: 10.1371/journal.pone.0155178 (PMC4873260; doi:10.1371/journal.pone.0155178)
Supplement: S2 Table — (DOCX) [file pone.0155178.s007.docx]

| **S2 Table. Patient Characteristics** | | | | | | | | | | |
| --- | --- | --- | --- | --- | --- | --- | --- | --- | --- | --- |
| Patient no. | 1 | 2 | 3 | 4 | 5 | 6 | 7 | 8 | 9 | 10 |
| Gender | F | M | M | M | M | F | M | M | F | F |
| Age (years) | 66 | 73 | 54 | 72 | 61 | 66 | 52 | 58 | 50 | 54 |
| Year first diagnosis | 2010 | 2010 | 2011 | 2007 | 2011 | 2007 | 2011 | 2007 | 2012 | 2011 |
| Primary tumour | Colon right | Sigmoid | Rectum | Sigmoid | Caecum | Rectum | Sigmoid | Rectum | Rectum | Sigmoid |
| Metastases | Pleura, lung, subcutaneous | Lnn^1^, liver, lung | Lnn, liver,  bone | Liver, Adrenal gland, soft tissue | Lnn, adrenal gland, peritoneal | Lnn, lung | Lnn, liver | Bone | Liver, lung, Lnn | Lung, liver |
| Prior therapy | FOLFOX, Irinotecan | CAPOX, Bevacizumab, Irinotecan | CAPOX, Bevacizumab, FOLFIRI | CAPOX, Bevacizumab, Irinotecan | FOLFOX, Irinotecan | CAPOX, Bevacizumab, CAPIRI | CAPOX, Bevacizumab, FOLFIRI | CAPOX, Irinotecan | CAPOX, Bevacizumab | FOLFOX, Bevacizumab, Irinotecan |
| BMI | 27,9 | 23,2 | 24,8 | 24,5 | 25,8 | 25,5 | 21,9 | 28,4 | 33 | 28,7 |
| RAS mutation analysis* | KRAS exon 2 / 3 | RAS WT^3^ | KRAS exon 2 / 3 | RAS WT | KRAS exon 2 / 3 | RAS WT | KRAS exon 2 / 3 | KRAS exon 2 / 3 | RAS WT | RAS WT |
| BRAF mutation analysis | BRAF WT | BRAF WT | BRAF WT | BRAF WT | BRAF mutated | BRAF WT | BRAF WT | BRAF WT | BRAF WT | BRAF WT |
| CT evaluation week 8** | PD | PD | SD | SD | PD | PD | PD | SD | PR | PR |
| Best Response on CT** | PD | PD | SD | SD | PD | PD | PD | SD | PR | PR |
| Progression Free Survival | 9 weeks | 8 weeks | 17 weeks | 33 weeks | 7 weeks | 7 weeks | 6 weeks | 23 weeks | 33 weeks | 26 weeks |
| Medical History | DM2^2^, hypertension | DM2, hypertension | urothelial cell carcinoma | Harmartoom, aneurysm a. iliaca right | - | - | - | hypercholesterolemia, hypothyroidism | cervical carcinoma, gastritis | DM2, pulmonary embolism |
| Psychiatric disease | - | - | - | - | - | - | - | - | - | - |
| *For some patients the quality of tissue was insufficient for the entire HRM-sequencing panel, in that case only KRAS exon 2 / 3 are tested **According to RECIST version 1.1 on CT evaluation  ^1^Lnn: lymph nodes ^2^DM2: Diabetes mellitus type 2 ^3^RAS WT: KRAS and NRAS exon 2-4 wild-type | | | | | | | | | | |
