# Supplementary material for: Early 18F-FDG PET/CT Evaluation Shows Heterogeneous Metabolic Responses to Anti-EGFR Therapy in Patients with Metastatic Colorectal Cancer
Source: PLoS One. 2016 May 19;11(5):e0155178. doi: 10.1371/journal.pone.0155178 (PMC4873260; doi:10.1371/journal.pone.0155178)
Supplement: S3 Table — (DOCX) [file pone.0155178.s008.docx]

| **S3 Table. Quantitative data** | |  |  |  |  |  |  |  |  |
| --- | --- | --- | --- | --- | --- | --- | --- | --- | --- |
| **Metabolically Most Active Lesion** | | | | | | | | | |
|  | SULpeak Pre | SULpeak Post | SULpeak % change | SULmax Pre | SULmax Post | SULmax % change | TLG Pre | TLG Post | TLG % change |
| 2 | 5,9 | 6,2 | 4,9 | 7,1 | 7,3 | 2,8 | 344,6 | 713,0 | 106,9 |
| 3 | 6,3 | 5,0 | -20,9 | 8,0 | 5,8 | -27,5 | 219,1 | 164,1 | -25,1 |
| 4 | 13,4 | 7,8 | -41,3 | 15,3 | 9,6 | -37,5 | 2754,2 | 687,9 | -75,0 |
| 5 | 5,4 | 4,5 | -17,6 | 7,2 | 5,2 | -27,8 | 59,2 | 83,0 | 40,2 |
| 6 | 3,1 | 4,3 | 36,9 | 4,1 | 5,6 | 35,5 | 7,8 | 10,9 | 39,3 |
| 7 | 11,4 | 11,5 | 0,4 | 12,9 | 13,1 | 1,9 | 621,2 | 1078,6 | 73,6 |
| 8 | 6,5 | 3,8 | -42,4 | 7,4 | 4,5 | -39,7 | 612,1 | 308,0 | -49,7 |
| 9 | 8,2 | 3,8 | -53,1 | 9,7 | 4,8 | -50,5 | 805,3 | 110,1 | -86,3 |
| 10 | 5,2 | 2,9 | -45,2 | 5,8 | 3,4 | -41,8 | 185,8 | 21,5 | -88,4 |
| **The sum of ≤ 5 target lesions (2 per tissue type)** | | | | | | | | | |
|  | SULpeak Pre | SULpeak Post | SULpeak % change | SULmax Pre | SULmax Post | SULmax % change | TLG Pre | TLG Post | TLG % change |
| 2 | 15,2 | 15,9 | 4 | 19,0 | 19,7 | 4 | 596 | 848 | 42 |
| 3 | 20,8 | 15,1 | -27 | 25,3 | 18,3 | -28 | 499 | 265 | -47 |
| 4 | 41,4 | 21,8 | -47 | 51,2 | 26,5 | -48 | 3582 | 786 | -78 |
| 5 | 10,8 | 8,6 | -20 | 13,6 | 10,2 | -25 | 91 | 108 | 19 |
| 6 | 3,1 | 4,3 | 37 | 4,1 | 5,6 | 36 | 8 | 11 | 39 |
| 7 | 27,0 | 24,1 | -11 | 31,1 | 28,5 | -9 | 1008 | 1382 | 37 |
| 8 | 6,5 | 3,8 | -42 | 7,4 | 4,5 | -40 | 612 | 308 | -50 |
| 9 | 18,2 | 9,9 | -45 | 22,1 | 12,4 | -44 | 875 | 130 | -85 |
| 10 | 13,1 | 6,4 | -51 | 15,5 | 7,5 | -52 | 211 | 31 | -85 |
| **The sum of all target lesions** | | | | | | | | | |
|  | SULpeak Pre | SULpeak Post | SULpeak % change | SULmax Pre | SULmax Post | SULmax % change | TLG Pre | TLG Post | TLG % change |
| 2 | 37,6 | 39,2 | 4 | 45,8 | 46,0 | 0 | 984 | 1177 | 20 |
| 3 | 27,0 | 19,7 | -27 | 32,9 | 23,4 | -29 | 543 | 278 | -49 |
| 4 | 41,4 | 21,8 | -47 | 51,2 | 26,0 | -49 | 3582 | 786 | -78 |
| 5 | 10,8 | 8,6 | -20 | 13,6 | 10,2 | -25 | 91 | 108 | 19 |
| 6 | 3,1 | 4,3 | 37 | 4,1 | 5,6 | 36 | 8 | 11 | 39 |
| 7 | 63,6 | 51,7 | -19 | 73,2 | 61,2 | -16 | 1357 | 1661 | 22 |
| 8 | 6,5 | 3,8 | -42 | 7,4 | 4,5 | -40 | 612 | 308 | -50 |
| 9 | 18,2 | 9,9 | -45 | 22,1 | 12,4 | -44 | 875 | 130 | -85 |
| 10 | 13,1 | 6,4 | -51 | 15,5 | 7,5 | -52 | 211 | 31 | -85 |
| Pre: baseline ^18^F-FDG PET Post: ^18^F-FDG PET after 2 cycles of cetuximab (4 weeks) | | | | |  |  |  |  |  |
